# Interaction patterns of methoprene-tolerant and germ cell-expressed *Drosophila* JH receptors suggest significant differences in their functioning

**DOI:** 10.3389/fmolb.2023.1215550

**Published:** 2023-08-15

**Authors:** M. Kolonko-Adamska, A. Zawadzka-Kazimierczuk, P. Bartosińska-Marzec, W. Koźmiński, G. Popowicz, A. Krężel, A. Ożyhar, B. Greb-Markiewicz

**Affiliations:** ^1^ Department of Biochemistry, Molecular Biology and Biotechnology, Wroclaw University of Science and Technology, Wroclaw, Poland; ^2^ Biological and Chemical Research Centre, Faculty of Chemistry, University of Warsaw, Warsaw, Poland; ^3^ Helmholtz Zentrum München, Neuherberg, Germany; ^4^ Bavarian NMR Center, Department of Chemistry, Technical University of Munich, Garching, Germany; ^5^ Department of Chemical Biology, Faculty of Biotechnology, University of Wrocław, Wrocław, Poland

**Keywords:** methoprene tolerant, germ cell expressed, *Drosophila melanogaster*, juvenile hormone, hormone receptor, structure–function

## Abstract

Methoprene-tolerant (Met) and germ cell-expressed (Gce) proteins were shown to be juvenile hormone (JH) receptors of *Drosophila melanogaster* with partially redundant functions. We raised the question of where the functional differentiation of paralogs comes from. Therefore, we tested Met and Gce interaction patterns with selected partners. In this study, we showed the ability of Gce and its C-terminus (GceC) to interact with 14-3-3 in the absence of JH. In contrast, Met or Met C-terminus (MetC) interactions with 14-3-3 were not observed. We also performed a detailed structural analysis of Met/Gce interactions with the nuclear receptor fushi tarazu factor-1 (Ftz-F1) ligand-binding domain. We showed that GceC comprising an Ftz-F1-binding site and full-length protein interacts with Ftz-F1. In contrast to Gce, only MetC (not full-length Met) can interact with Ftz-F1 in the absence of JH. We propose that the described differences result from the distinct tertiary structure and accessibility of binding sites in the full-length Met/Gce. Moreover, we hypothesize that each interacting partner can force disordered MetC and GceC to change the structure in a partner-specific manner. The observed interactions seem to determine the subcellular localization of Met/Gce by forcing their translocation between the nucleus and the cytoplasm, which may affect the activity of the proteins. The presented differences between Met and Gce can be crucial for their functional differentiation during *D. melanogaster* development and indicate Gce as a more universal and more active paralog. It is consistent with the theory indicating *gce* as an ancestor gene.

## Background

The fundamental mechanisms and pathways responsible for the development of organisms were shown to be preserved during evolution. For this reason, *Drosophila melanogaster* has been established as an important model organism to study how gene expression is regulated to enable the transition from a single cell to an embryo and, finally, to a mature organism (Jennings, 2011). In contrast to higher organisms, the growth and development of insects are controlled by two major physiologically active non-peptide hormones: the steroid hormone 20-hydroxyecdysone (20E) and the sesquiterpenoid juvenile hormone (JH) (Nijhout, 1994). In addition, these hormones perform key functions during insect adult life, including reproduction, pheromone production, migration, and diapause (Wyatt and Davey, 1996; Gruntenko and Rauschenbach, 2008). The mechanism of JH signaling was not explained for a long time (Dubrovsky, 2005). Finally, the methoprene-tolerant (Met) protein was discovered during the screening of *Drosophila* mutants resistant to JH analogs and proposed as a JH receptor (Wilson and Fabian, 1986; Shemshedini and Wilson, 1990; Ashok et al., 1998; Charles et al., 2011). Surprisingly, while the *met* gene deletion was lethal for most insect species, the *Drosophila* mutant was alive. This discrepancy was explained by the discovery of the Met paralog, the germ cell-expressed (Gce) protein, which ensured the mutants’ survival (Baumann, 2010a; Jindra et al., 2015). Currently, both paralogs are considered *Drosophila* JH receptors (Jindra et al., 2015; Zhu, 2022). Interestingly, most studied insect species have a single JH receptor presenting higher sequence similarity to Gce than to Met (Wang et al., 2007): *Tribolium castaneum* possesses a single Met-like ortholog, while *Aedes aegypti, Culex pipiens*, and *Anopheles gambiae* have Gce-like single homologs (Wang et al., 2007). The *met* gene has been suggested to be a product of the duplication of the ancestral *gce* gene during the origination of *drosophilids* (Wang et al., 2007).

Met and Gce belong to the basic helix–loop–helix/Per–Arnt–Sim (bHLH-PAS) transcription factor (TF) family (Ashok et al., 1998; Moore et al., 2000), which is known to regulate important developmental and physiological processes in eukaryotes (Kewley et al., 2004). Met and Gce present a domain structure typical for bHLH-PAS TFs (Michael and Partch, 2013). The N-terminally-located bHLH domain is responsible for dimerization and DNA binding, while the following PAS-1 domain specifies dimerization partners to activate specific genes. Centrally located, PAS-2 is a sensor domain binding a ligand/receiving signal, leading to the conformational changes and transfer of the signal (Kewley et al., 2004). The bHLH-PAS proteins are known to function as homo- or heterodimers with the prevalence of the latter (Crews, 2003). In fact, Met and Gce form heterodimers (and homodimers in the case of Met) (Godlewski et al., 2006), which dissociate upon JH binding (Godlewski et al., 2006). Therefore, if the JH concentration is high, both Met and Gce act as monomers interacting with steroid-hormone coactivator Taiman (TAI) in *Drosophila* and its homologs in other species, e.g., βFtz-F1-interacting steroid receptor coactivator (FISC) in *Aedes aegypti* and steroid receptor coactivator (SRC) in *Tribolium castaneum* (Ashok et al., 1998; Li et al., 2011; Zhang et al., 2011). The JH-Met-TAI complex binds to the juvenile hormone response element (JHRE) and activates the transcription of the early trypsin gene (Li et al., 2011).

Although both Met and Gce were shown to prevent precocious differentiation during metamorphosis in insects (Parthasarathy, Tan, and Palli, 2008), their functions are only partially redundant, and each protein presents its own tissue and developmental stage-specific expression (Abdou et al., 2011). For example, Met ensures the proper development of eye tissue and genitals (Baumann et al., 2010b), while Gce is responsible for the development of the digestive system and induction of E75A nuclear receptor gene expression, the product of which is essential during larval metamorphosis (Dubrovsky et al., 2011).

In contrast to bHLH and PAS domains presenting high sequence homology (78% for bHLH, 68% for PAS-1, and 86% for PAS-2) (Moore et al., 2000), the C-termini of Met and Gce (MetC and GceC, respectively) are highly differentiated (Supplementary Figure S1, based on the SIM server (Huang and Miller, 1991)) (Moore et al., 2000; Kolonko et al., 2016; Kolonko et al., 2020). Variable C-terminal fragments of bHLH-PAS proteins were shown to comprise transactivation domains (TADs, as described by Moffett and Pelletier (2000)) and are considered important elements modulating protein activity (Kewley et al., 2004; Furness et al., 2007). For example, MetC was proposed to contribute to the specificity of JH III binding to the heterodimers Met/Cyc (cycle) (Shin et al., 2012). Knowing that the most significant differences between Gce and Met sequences occur between their C-terminal fragments (MetC and GceC; Supplementary Figure S1), we focused on protein partners interacting (or predicted to interact) exactly with these areas. As the first studied protein, we chose the orphan nuclear receptor fushi tarazu factor-1 (Ftz-F1) (Bernardo and Dubrovsky, 2012a). In the absence of JH, Ftz-F1 participates in the induction of metamorphosis regulated by 20E (Bernardo and Dubrovsky, 2012b). When the JH concentration is high, Ftz-F1 enables the expression of specific JH-dependent genes by binding to Met or Gce (Bernardo and Dubrovsky, 2012b). Met and Gce were shown to interact with the Ftz-F1 ligand-binding domain (LBD) in a unique way by hydrophobic contact of a novel nuclear receptor box (NR-box, LIXXLL sequence) located in their C-termini. The Ftz-F1 LBD is composed of 12 α-helixes, forming a canonical charge clamp (activation function 2 (AF2)) (Yoo et al., 2011; Bernardo and Dubrovsky, 2012a). Interestingly, an additional interaction site located in the Met C-terminus (Q_R_ region) was shown to be indispensable for interaction stabilization (Bernardo and Dubrovsky, 2012a). Verifying the basis for the differences in Met and Gce binding, we performed a detailed structural analysis of MetC, GceC, and peptides corresponding to the NR-boxes of MetC or GceC (Met^PEP^ and Gce^PEP^, respectively) during interaction with the Ftz-F1 LBD.

The functional differentiation of Met and Gce can be partially related to distinct patterns of the nuclear localization and nuclear export signals (NLSs and NESs, respectively), resulting in differentiated regulation of shuttling (Greb-Markiewicz et al., 2011; 2015). For example, the C-terminally-located NLS (Supplementary Figure S1), which acts synergistically with the NLS in the PAS-2 domain and is activated by JH binding, is a unique characteristic of Gce. On the other hand, Gce lacks a dominant NLS located in the PAS-1 domain of Met (Greb-Markiewicz et al., 2011; Greb-Markiewicz et al., 2015). In a previous study, we observed that in contrast to Met, wild-type Gce was evenly located in the analyzed COS-7 cells in the absence and presence of JH (Greb-Markiewicz et al., 2015). Since we expected Gce to transfer to the nucleus in the presence of JH, we asked about factors influencing localization. We performed interaction prediction and identified S462 and S670 located in the C-terminus of Gce as a part of putative 14-3-3 protein-binding sites (Scansite 2.0 (Obenauer et al., 2003), Supplementary Table S1) (Greb-Markiewicz et al., 2015). The 14-3-3 protein family members are ubiquitously expressed in all eukaryotic cells and present high aa sequence conservation among organisms, ranging from yeast to mammals (Darling et al., 2005). 14-3-3 proteins participate in signal transduction, and a wide range of their interactions allows 14-3-3 to regulate important processes, such as control of the cell cycle, apoptosis, development of the nervous system, and neurodegeneration (Obsilova et al., 2014). The interaction with 14-3-3 can affect the partner protein structure, stability, and subcellular localization (Obsilova et al., 2014). As shown previously, S462A and/or S670A Gce mutants were localized in the nucleus of COS-7 cells in the presence of JH. These results suggested that in the wild-type GceC, NLS was inactive while being masked by 14-3-3 binding (Greb-Markiewicz et al., 2015). Interestingly, no 14-3-3-binding sites were predicted in the MetC sequence. We performed comprehensive interaction studies to test the hypothesis distinguishing Gce (or GceC) and Met (or MetC) in the context of 14-3-3 binding. Moreover, we performed preliminary fluorescence imaging experiments to detect fluctuations in the localization of the expressed proteins.

In this study, we present the results of the interaction analysis of MetC, GceC, and peptides corresponding to the novel NR-boxes localized in MetC or GceC (Met^PEP^ and Gce^PEP^) with the Ftz-F1 LBD (based on the study by Dubrovsky et al. (2011)). We also tested MetC and GceC interactions with 14-3-3. It is worth noting that overexpression in bacterial cells and purification of the full-length JH receptors (similar to other bHLH-PAS TFs) were not efficient; so, for *in vitro* studies, we used C-terminal fragments, which are considered responsible for interactions with both the Ftz-F1 LBD and 14-3-3. During nuclear magnetic resonance (NMR) studies, we performed two sets of experiments: MetC and GceC ^15^N-HSQC spectra were recorded in the absence and presence of selected partners, and Ftz-F1 LBD ^15^N-HSQC spectra were measured in the absence and presence of MetC/GceC or peptides Met^PEP^ and Gce^PEP^. The dissociation constant (*K*_d_) of the Ftz-F1 LBD and Met^PEP^ was determined by isothermal titration calorimetry (ITC). Finally, a pull-down assay combined with fluorescence imaging was used to analyze the full-length Met and Gce, as well as MetC and GceC interactions, and to determine fluctuations in their subcellular localization forced by these interactions. Furthermore, more detailed studies of the effects of interactions on protein distribution are currently being carried out.

All the presented results are consistent and indicate distinct interaction patterns of the two analyzed *Drosophila* JH receptors. As shown, GceC and full-length Gce interact with the Ftz-F1 LBD since the C-terminus of the protein, comprising the NR-box, is easily accessible for a partner. In the case of Met, only the C-terminal domain (MetC) can interact with Ftz-F1, suggesting that the full-length protein C-terminally-located NR-box is hidden in the protein structure and unavailable. Only Gce and GceC interact with the 14-3-3 protein, which probably affects its subcellular localization and, thus, activity. Moreover, we observed different patterns of chemical shifts in GceC spectra in the presence of the Ftz-F1 LBD or 14-3-3. We assume that the determined specific interactions can force MetC and GceC to change the structure, depending on the interaction partner and determining specific receptor activity. The presented research is the first attempt to explain why *Drosophila* Gce is only partially redundant with Met and why the roles of these two proteins could differ. Furthermore, more detailed research is still needed, and we believe that our study could serve as a starting point directing future attempts.

## Materials and methods

### Preparation of expression vectors and the overexpression of MetC

The cDNA encoding the full-length *D. melanogaster* Met protein (Q9VYW2) was provided by T. Godlewski (Prof. Thomas G. Wilson, Ohio State University). The MetC sequence (509–716 aa) was amplified using polymerase chain reaction (PCR) and the primers forward 5′ att aat tcc atg gCG GGC CGG CAA AAG GTG 3′ and reverse 5′ a tta agc ggc cgc TCA TCG CAG CGT GTC 3′, thus introducing a restriction site for NcoI and NotI, respectively (underlined). The obtained PCR product was digested with suitable enzymes and cloned into a pETH-SUMO vector (EMBL, Germany) in the reading frame with 6 × His and SUMO tags. The presence of the insert in the vector was confirmed by sequencing.

The BL21(DE3) *E*. *coli* cells (Novagen) were transformed with a pETH-SUMO vector containing 6 × His-SUMO-MetC cDNA and plated on lysogeny broth (LB) agar containing 50 μg/ml of kanamycin. After overnight incubation at 37°C, a single colony was used to inoculate 20 ml of the LB medium containing 50 μg/ml of kanamycin. The bacterial culture was incubated overnight in a rotary shaker (220 rpm) at 37°C. Then, 10 ml of the overnight starting culture was used to inoculate 500 ml of the N-5052 medium with 100 μg/ml of kanamycin (for unlabeled samples and samples labeled with N^15^; in the case of N^15^ and C^13^ labeling, minimal M9 medium was used). The cell cultures were incubated at 37°C until the optical density (OD_600_) reached 1.5. Then, incubation was continued at 20°C for 15 h (in the case of the M9 medium, IPTG, in a final concentration of 0.2 mM, was added). Finally, the cells were harvested by centrifugation at 4,000 × *g* (10 min, 4°C), resuspended in lysis buffer (20 mM Tris-HCl pH 7.5, 100 mM NaCl, 20 μg/ml DNase I and RNase A, 0.2 mg/ml PMSF, and 1 mM β-mercaptoethanol (βME)), and disrupted by sonication (10 min, 60%, 30 s/30 s). After lysate centrifugation (20,000 rpm, 1 h, at 4°C), the soluble fraction containing 6 × His-SUMO-MetC was collected. Immobilized metal affinity chromatography (IMAC) on the Ni^2+^-NTA resin was chosen as the first purification step. The unbound proteins were washed away with lysis buffer, and the 6 × His-SUMO-MetC protein was digested with SUMO hydrolase dtUD1 (EMBL, Germany) (Weeks et al., 2007) on the column. Finally, tag-less MetC was collected as a flow-through, concentrated to 2 ml on the Amicon Ultra Centrifugal Filter (Millipore) with a cutoff of 10 kDa, and loaded on a Superdex 200 10/300 GL column (Amersham Biosciences) connected to ÄKTA avant (GE Healthcare). For MetC NMR analysis, 20 mM Bis-Tris pH 6.5, 150 mM NaCl, and 5 mM βME were used at the buffer. Samples of the purified MetC protein were collected for further analysis.

### cDNA vector preparation and the overexpression of GceC and Ftz-F1 LBD

Cloning and the expression of GceC and the Ftz-F1 LBD were described in detail previously (Kolonko et al., 2020). The cDNA encoding the *D. melanogaster* Gce protein (UniProt accession number: Q9VXW7) was provided by Prof. Thomas G. Wilson (Ohio State University) and used as a template for PCR. The amplified GceC fragment (661–959 aa) was cloned into the pET-M11 vector. Next, the cDNA encoding the Ftz-F1 LDB (P33244, 786–1027 aa) was synthesized (Gene Art Thermo Fisher Scientific) and cloned into the pET-15b vector. Both vectors introduced a 6 × His tag at the protein N-terminus.

GceC and Ftz-F1 LBD expression procedures were analogous to the procedure described for MetC. The obtained cell pellets were resuspended in lysis buffer (20 mM Tris-HCl pH 7.5, 100 mM NaCl, 20 μg/ml DNase I and RNase A, 0.2 mg/ml PMSF, and 1 mM β-mercaptoethanol (βME)) and disrupted by sonication (10 min, 60%, 30 s/30 s), followed by centrifuging at 20,000 rpm for 1 h at 4°C.

In the case of GceC, the protein was present in the sediment fraction, so the protein was purified under denaturing conditions. After denaturation with 6 M GdmCl_2_ and renaturation by 100 x dilution in refolding buffer (20 mM Tris-HCl pH = 8.0, 7.5% glycerol, and 5 mM DTT), the protein was bound to the Ni^2+^-NTA resin, eluted with 0.35 M imidazole, and loaded on the Superdex 200 10/300 GL column (Amersham Biosciences) equilibrated with GceC NMR buffer (PBS pH = 7.4, 2 mM DTT). Samples containing the purified GceC protein were collected.

The Ftz-F1 LBD was present in the soluble fraction. IMAC on Ni^2+^-NTA was used as the first step of the purification procedure. The unbound proteins were washed away with wash buffer (20 mM Tris-HCl pH 7.5 and 100 mM NaCl), and the 6 × His-Ftz-F1 protein was eluted with 0.5 M imidazole. The collected fusion protein was digested overnight with thrombin (50 U for 10 mg of 6 × His-Ftz-F1, Sigma-Aldrich), concentrated to 2 ml on the Amicon Ultra Centrifugal Filter (Millipore) with a cutoff of 10 kDa, and loaded on the Superdex 200 10/300 GL column (Amersham Biosciences) equilibrated with a suitable buffer (Ftz-F1 LBD NMR buffer: 20 mM sodium phosphate pH 6.8, 50 mM NaCl, 1 mM TCEP, and 0.5 mM EDTA). Samples containing the purified Ftz-F1 LBD protein were collected.

### GceC phosphorylation in bacterial cells

To obtain the GceC protein in phosphorylated form (P-GceC), its overexpression was carried out in the presence of a protein kinase A catalytic subunit (PKA) with S/T phosphorylation activity (Plasmid #14921, Addgene) (Narayana et al., 1997). The BL21(DE3) *E*. *coli* cells (Novagen) were co-transformed with the pET-M11 plasmid encoding 6 × His-GceC and the pET-15b plasmid encoding PKA and plated on LB agar containing 50 μg/ml kanamycin and 50 μg/ml ampicillin (resistance to ampicillin was introduced with the pET-15b vector). The following protein expression and purification steps were the same as for the non-phosphorylated GceC control since the PKA was present only in the soluble fraction.

### cDNA vector preparation and the expression of the 14-3-3 protein

The full-length 14-3-3 human isoform ζ, cDNA (P63104) used for tests was provided by Dr. Marek Orłowski (Wroclaw University of Science and Technology) and amplified (PCR) with the primers, forward 5′ aaa acc atg gcc ATG GAT AAA AAT 3′ and reverse 5′ aaa agc ggc cgc TTA ATT TTC CCC TCC 3’, thus introducing restriction sites for NcoI and NotI, respectively (underlined). The digested product was cloned into a pET-M11 vector in a reading frame with a 6 × His tag. The correctness of the obtained pET-M11-6 × His-14-3-3 vector was confirmed by sequencing.

The protein expression procedure was the same as for MetC. The obtained cell pellet was resuspended in lysis buffer and disrupted by sonication (10 min, 60%, 30 s/30 s), followed by centrifugation (20,000 rpm, 1 h, at 4°C). The soluble fraction containing 6 × His-14-3-3 was loaded on the Ni^2+^-NTA resin. Next, 6 × His-14-3-3 was eluted with the buffer supplemented with 0.5 M imidazole, concentrated to 2 ml on the Amicon Ultra Centrifugal Filter (Millipore) with a cutoff of 10 kDa, and loaded on the Superdex 75 10/300 GL column (Amersham Biosciences) equilibrated with the appropriate buffer. Fractions containing purified 14-3-3 were collected.

### Synthesis of peptides

The lyophilized 19-residue MetC and GceC peptides (Met^PEP^, LERIVLYLIENLQKSVDSA; Gce^PEP^, LEKAVLRLIQNLQKSGENG) representing the novel NR-box were synthesized (PSL GmbH, Heidelberg). The product purity determined by NMR was >98%. The peptides were finally dissolved in appropriate buffers.

### Sodium dodecyl sulfate–polyacrylamide gel electrophoresis

Samples collected during protein expression and purification were analyzed by sodium dodecyl sulfate–polyacrylamide gel electrophoresis (SDS-PAGE) (12% polyacrylamide gels developed in a Tris/glycine system (Laemmli, 1970)). The Precision Plus Protein™ Standards Weight Marker (Bio-Rad) was used as a molecular mass (MM) protein standard. The gels were stained with SimplyBlue™ SafeStain (Invitrogen).

### NMR analysis

The NMR spectra of MetC (for resonance assignment and chemical shift perturbation experiment), as well as the NMR spectra of Ftz-F1 LBD (for resonance assignment transfer), were collected using the Bruker Avance III HD spectrometer with a proton frequency of 800 MHz, equipped with a 5 mm TCI-HCN cryogenically cooled probe. GceC, as well as Ftz-F1 LBD spectra for the chemical shift perturbation experiment, was measured in the absence and presence of selected partners (Ftz-F1 or 14-3-3 for GceC and MetC, GceC, Met^PEP^, or Gce^PEP^ for the Ftz-F1 LBD) using the Bruker Avance III HD spectrometer with a proton frequency of 600 MHz, equipped with a 3 mm cryoprobe and automatic sample changer (SampleJet). All the protein samples for NMR measurements were prepared in the appropriate buffer (20 mM Bis-Tris pH 6.5, 150 mM NaCl, and 5 mM βME for MetC; PBS pH 7.4 and 2 mM DTT for GceC; and 20 mM sodium phosphate pH 6.8, 50 mM NaCl, 1 mM TCEP, and 0.5 mM EDTA for Ftz-F1 LBD), with the addition of 10% D_2_O to provide a lock signal, and measured at defined temperatures (18°C for MetC and GceC, and 29°C for Ftz-F1 LBD). The conditions of MetC measurements were optimized to slow down the chemical exchange of amide protons, which, under the initial conditions, led to the disappearance of many peaks in the ^15^N-HSQC spectrum. For Ftz-F1, the conditions were set identically to the ones in the initial resonance assignment (Daffern et al., 2019). The conditions of GceC were optimized to avoid protein precipitation. The interaction and titration analysis were performed using ^15^N-HSQC experiments.

The resonance assignment of MetC was performed using a uniformly isotopically labeled (^13^C and ^15^N) sample with a concentration of 450 μM. A set of multidimensional experiments was utilized, including 3D HNCO, 5D HN(CA)CONH (Kazimierczuk et al., 2010), 5D (HACA)CON(CA)CONH (Zawadzka-Kazimierczuk et al., 2012b), and 5D HabCabCONH (Kazimierczuk et al., 2010). All these experiments were acquired using nonuniform sampling of the evolution time space. They were processed using the signal separation algorithm for 3D (Stanek and Koźmiński, 2010) and 5D (Kosiński et al., 2017) data. For sparse processing of 5D datasets (Kazimierczuk et al., 2010), 3D HNCO was used as a basis spectrum. Spectra were analyzed using NMRFAM-SPARKY software (Lee et al., 2015). The resonance assignment was supported by the TSAR (Zawadzka-Kazimierczuk, 2012a; Piai et al., 2016). The experimental parameters are given in Supplementary Table S2. The MetC chemical shifts were deposited in the BMRB (Hoch et al., 2023), under accession number 51720.

The chemical shift perturbation experiments of MetC upon adding Ftz-F1 LBD were performed using the ^15^N-HSQC technique. The titration scheme was adapted from the study by Sekhar et al. (2017); the two initial samples were prepared (sample A with 150 μM MetC concentration and with no Ftz-F1 and sample B with 150 μM MetC concentration and 500 μM Ftz-F1 LBD concentration). The Ftz-F1 LBD concentration was changed by cross-mixing (simultaneously exchanging equal amounts of the solution between the samples) so that the sample volumes and MetC concentration remained constant. The measurement was performed for the following concentrations of the Ftz-F1 LBD: 0, 40, 82, 122, 199, 301, and 500 μM. The dissociation constant was calculated according to the published protocol (Williamson, 2013).

For the Ftz-F1 LBD, the resonance assignment (of the peaks showing changes in the position or intensity on binding to the Met^PEP^ and Gce^PEP^ peptides) was transferred from the study by Daffern et al. (2019). The transfer was supported with 3D HNCO (Kay et al., 1990), HN(CA)CO (Clubb et al., 1992), HN(CO)CA (Bax and Ikura, 1991), HNCA (Kay et al., 1990), and CBCA(CO)NH (Grzesiekt and Bax, 1992) spectra. The sample used for these experiments was uniformly ^13^C,^15^N-labeled, and its concentration was 450 μM. The 3D HNCO experiment was recorded using nonuniform sampling of the evolution time space, while all other experiments were performed conventionally. The experimental parameters are given in Supplementary Table S3.

The chemical shift perturbation experiments of GceC upon adding Ftz-F1 LBD or 14-3-3 and FTZ-F1 LBD upon adding MetC, GceC, Met^PEP^, or Gce^PEP^ were performed using the ^15^N-HSQC technique. The 100 μM ^15^N-labeled GceC spectra were recorded in the absence and presence of the equimolar amount of unlabeled Ftz-F1 LBD or 14-3-3. The 150 μM ^15^N-labeled FTZ-F1 LBD spectra were recorded in the absence and presence of an equimolar quantity of unlabeled MetC, GceC, Met^PEP^, or Gce^PEP^. Finally, the peptide concentration was used up to the solubility limit (approximately 3 mM for Met^PEP^ and 2 mM for Gce^PEP^).

### Isothermal titration calorimetry

The binding of Met^PEP^ to the Ftz-F1 LBD was monitored using a Nano Isothermal Titration Calorimeter (ITC) (TA Waters, USA) at 25°C with a cell volume of 1 ml. All experiments were performed in duplicate, in 20 mM HEPES buffer, pH 6.8, 150 mM NaCl, and 1 mM TCEP. The titrate (Ftz-F1 LBD) concentration was 0.035 mM, whereas the titrant (Met^PEP^) concentration was 1.06 mM. After system equilibration, 20 injections of the titrant were made into the reaction cell in 12.5 µl increments at 300 s intervals with stirring at 200 rpm. To determine the heats of titrant dilution, control titrations were performed by identical injections of Met^PEP^ to the buffer. The net reaction heat was calculated by subtracting the dilution heat from the corresponding total heat of the reaction. The titration data were analyzed using NanoAnalyze (version 3.3.0), NITPIC (version 2.0.7) (Scheuermann and Brautigam, 2015), and SEDPHAT (version 15.2b) (Houtman et al., 2007). Data were fitted to an independent model A + B ⇌ AB using the global fitting mode. The error estimates for the fitting results were produced by Monte Carlo analysis with 1,000 iterations and confidence levels of 0.7.

### Preparation of cDNA vectors for protein expression in mammalian cells

It is worth mentioning that in previous studies (Greb-Markiewicz et al., 2011; Greb-Markiewicz et al., 2015) the Gce isoform (provided by Prof. T Wilson and T. Godlewski, Ohio State University) comprising 689 aa was used. Later, this isoform was substituted by a new isoform containing an additional N-terminus 270 aa long (UniProt accession number Q9VXW7). However, according to Baumann (2010a), both Gce isoforms perform the same functions, and the first 270 amino acid residues do not affect the protein activity. In addition, no NLSs/NESs were predicted to influence localization in this region. Hence, for expression studies in mammalian cells, to compare results with those of previous studies, we also used the short isoform of GCE in this study.

cDNA fragments corresponding to Met, Gce, MetC, and GceC, with a C-terminally introduced FLAG peptide sequence (DYKDDDDK), were cloned into the multiple cloning site (MCS) of the pEYFP-C1 vector (Clontech) (pEYFP-Met-FLAG, pEYFP-Gce-FLAG, pEYFP-MetC-FLAG, and pEYFP-GceC-FLAG) with EcoRI and SalI restriction enzymes. The cDNA of the Ftz-F1 LBD and 14-3-3 was cloned into the pECFP-C1 vector (Clontech) (pECFP-Ftz-F1 LBD and pECFP-14-3-3, respectively) with XhoI and BamHI restriction enzymes. All the primer sequences are presented in Supplementary Table S4. All constructs were confirmed by DNA sequencing.

### Mammalian cell culture and transfection

All experiments were performed as described previously (Kolonko et al., 2020). In short, African green monkey kidney fibroblast COS-7 cells (ATCC CRL-1651) were grown in Ø6 cm plates at 37°C 24 h prior to transfection (Xfect Transfection Reagent Takara Bio) with 9 μg of the appropriate vector encoding the selected protein, or with 6 μg of each from two selected vectors in the case of co-transfection. pEYFP-C1 and pECFP-C1 vectors were used as controls. After transfection, the culture was further incubated for 24 h and used for fluorescence imaging.

### ANTI-FLAG pull-down assay

All experiments were performed as described previously (Kolonko et al., 2020). After 24 h of incubation, the cells were lysed (lysis buffer: 25 mM Tris-HCl pH 7.4, 150 mM NaCl, 1% NP-40, 1 mM EDTA, and 5% glycerol), transferred to a microcentrifuge tube, and centrifuged at 13,000 × *g* for 15 min. Importantly, in the case of 14-3-3 interaction assays dependent on partner phosphorylation, we used phosphatase inhibitors, 5 μM sodium orthovanadate, 10 μM sodium molybdate, 10 μM PMSF, and cOmplete™ EDTA-free Protease Inhibitor (cat. No. 11836170001, Roche) in lysis buffer. The soluble fractions were incubated on ice with 20 μl of EZview™ Red ANTI-FLAG M2 Affinity Gel (Sigma-Aldrich) for 2 h. The unbound proteins were washed away, and the gel was incubated with the elution buffer (50 mM Tris-HCl, pH 7.4, 150 mM NaCl, and 100 μg/ml FLAG peptide) for 30 min. The eluted fractions were collected for Western blot analysis.

### Western blot analysis

All experiments were performed as described previously (Kolonko et al., 2020). Briefly, samples obtained during the pull-down assay were separated by SDS-PAGE using 12% gels and transferred to the Whatman Protran nitrocellulose transfer membrane (Protran BA85, Schleicher & Schuell Pure, Sigma-Aldrich). The membranes were blocked with milk powder (Milchpulver, blotting grade, Roth), then incubated overnight with specific primary anti-GFP polyclonal antibodies (1:5,000, cat. no. SAB2702197, Sigma-Aldrich) or anti-14-3-3 antibodies (1:5,000, cat. no. AB9748-I, Millipore), followed by the incubation with secondary horse anti-mouse antibodies (for anti-GFP, 1:10,000, cat. no. PI-2000, Vector Laboratories) or goat (for anti-14-3-3, 1:10,000, Sigma-Aldrich, cat. no. A 6154) anti-mouse antibodies coupled to horseradish peroxidase. Signals were detected using the SuperSignal™ West Pico PLUS Substrate Chemiluminescent kit (Thermo Scientific™). Finally, the membranes were exposed to Kodak BioLight film. The MM of each fusion protein was calculated based on aa sequences using the ProtParam server (Gasteiger, 2019) (YFP-Met-FLAG 107 kDa, YFP-Gce-FLAG 105 kDa, YFP-MetC-FLAG 51.4 kDa, YFP-GceC-FLAG 64 kDa, CFP-Ftz-F1 LBD 55 kDa, and CFP-14-3-3 57 kDa).

### Fluorescence microscopy

The experiment was performed as described previously (Kolonko et al., 2020). Briefly, fluorescence microscopy was performed on an Olympus IX71 microscope with a CFP or YFP filter 24 h after transfection. Cells transfected with empty pEYFP-C1 and pECFP-C1 vectors were used as controls.

## Results

### Protein expression and purification

**FIGURE 1:**
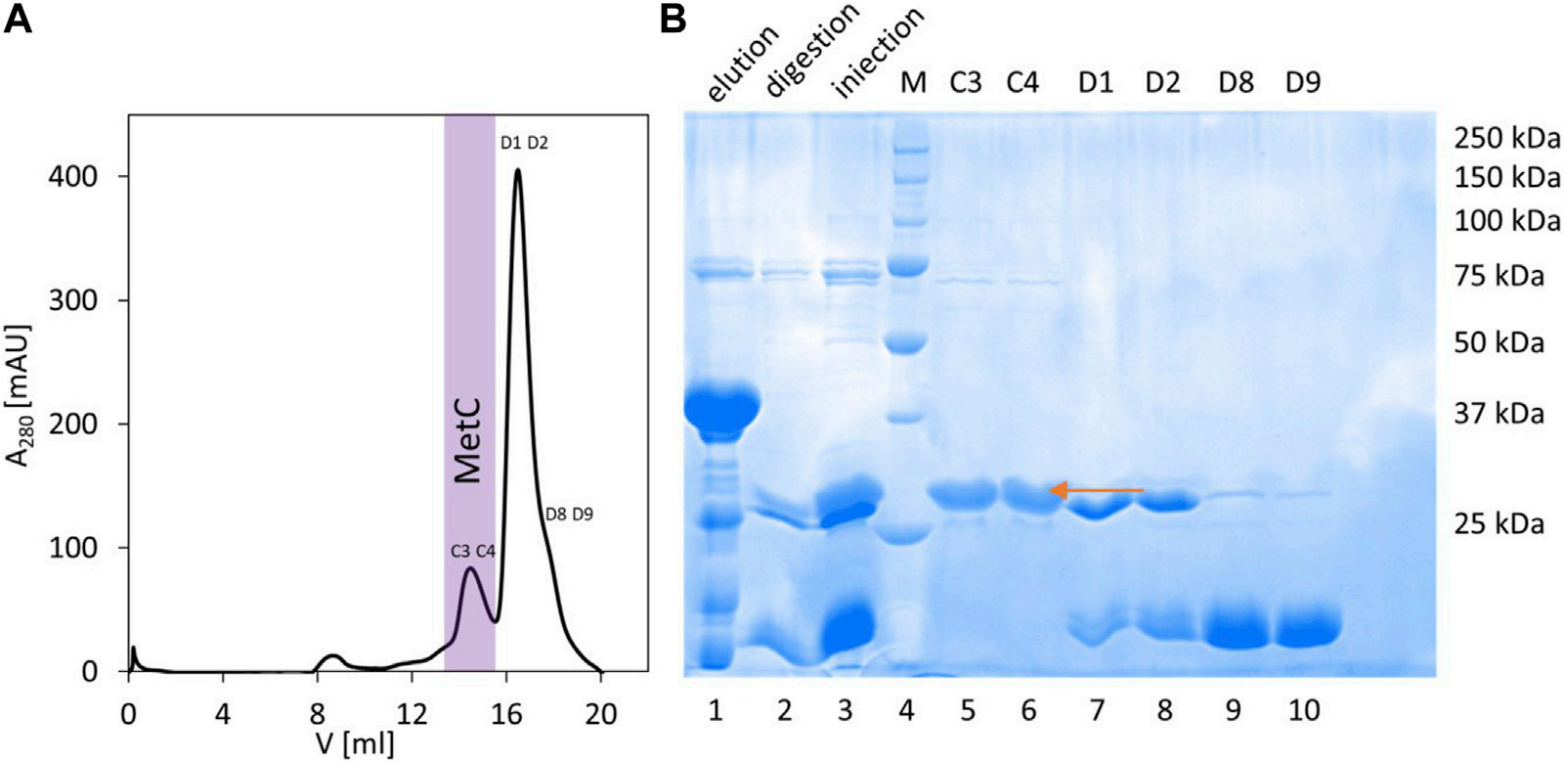
MetC purification. **(A)** Preparative SEC performed on the Superdex 200 10/300 GL column. Fraction numbers indicate fractions selected for SDS-PAGE. Fractions containing purified MetC are marked with purple. **(B)** SDS-PAGE analysis of the MetC samples. Lane 1: fractions eluted from the Ni^2+^-NTA resin; lane 2: protein after digestion with dTUd1 protease; lane 3: sample injected on the Superdex 200 column; lane 4: molecular mass standards; and lanes 5–10: fractions from SEC. The arrow indicates the MetC protein.

In the case of MetC, the previously developed protocol (expression from the pColdTM TF vector, Takara) (Kolonko et al., 2016) was not sufficient to obtain isotopically (^15^N and ^13^C) labeled samples. Thus, we used another vector, pET-SUMO (EMBL, Germany), introducing 6 × His and SUMO tags at the protein N-terminus. The SUMO tag acts both as a chaperonin and an initiator of protein folding and enhances the stability and solubility of target proteins (Jg et al., 2006). The expressed fusion protein was present in the soluble fraction, and the developed MetC purification procedure consisted of IMAC with a Ni^2+^-NTA resin, fusion protein digestion on a column with SUMO hydrolase (dTUd1, EMBL, Germany) (Weeks et al., 2007), and final purification combined with buffer exchange on the Superdex 200 10/300 GL column (Figure 1A, elution profile). As a result, the purified MetC was visible as a single band on the 12% SDS-PAGE gel (Figure 1B) with decreased electrophoretic mobility (approximately 29 kDa) compared to the MM of MetC (23.4 kDa) (Kolonko et al., 2016). Such behavior is a characteristic of IDPs (Tompa, 2002; Iakoucheva et al., 2009), binding less SDS due to the specific sequence composition.

GceC purification was described in detail previously (Kolonko et al., 2020). In short, the protein was insoluble under all tested conditions, so we decided to develop a purification procedure under denaturing conditions. After GceC denaturation with GdmCl_2_, the protein was refolded by dilution and bound to the Ni^2+^-NTA resin since GceC was expressed in fusion with the 6 × His tag. Finally, SEC on the Superdex 200 10/300 GL column was used to remove small impurities. Purified GceC electrophoretic mobility was decreased similar to MetC: its electrophoretic mobility corresponded to the protein with an MM of 42 kDa instead of the expected 36 kDa. GceC belongs to IDPs (Kolonko et al., 2020).

**FIGURE 2:**
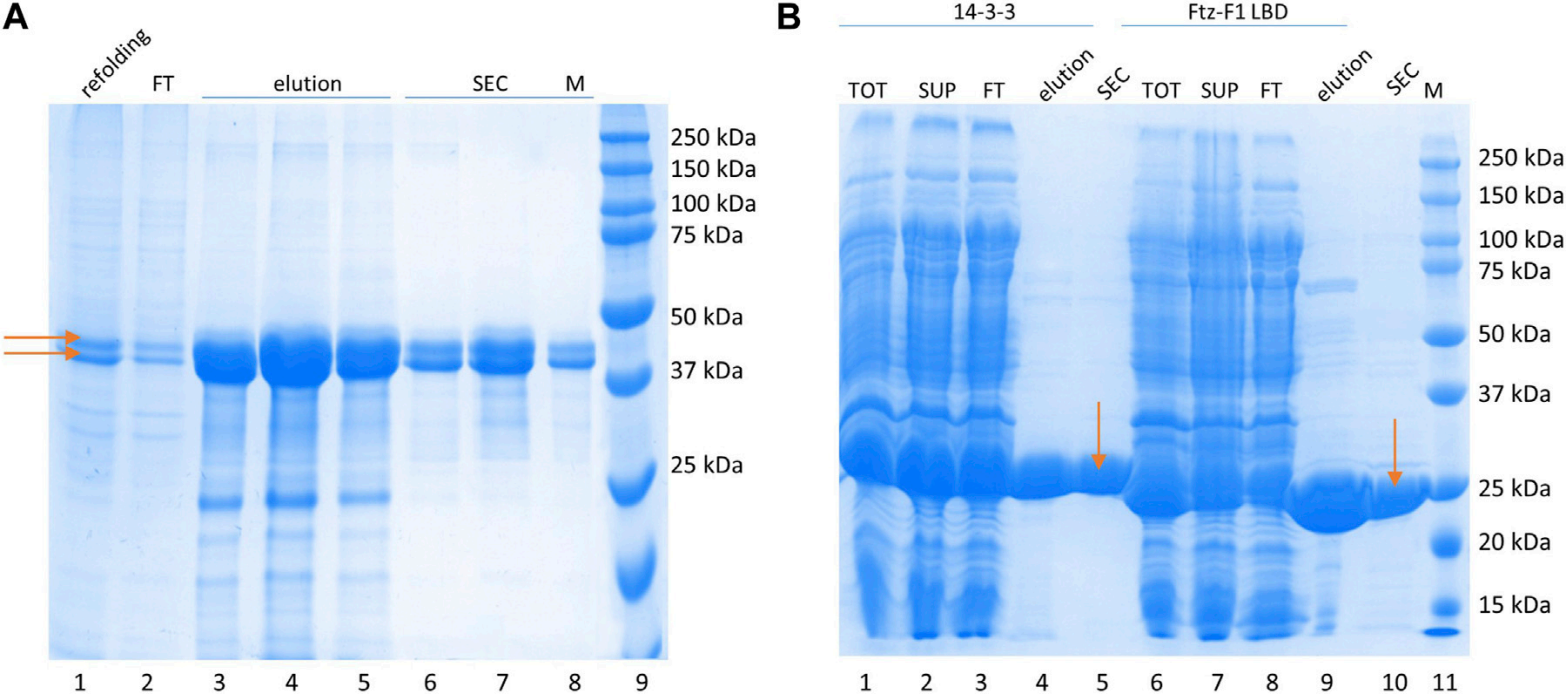
Protein purification. **(A)** P-GceC purification. SDS-PAGE analysis of the P-GceC samples. Lane 1: refolded proteins; lane 2: proteins not bound to the Ni^2-^-NTA resin; lanes 3–5: fractions eluted from the resin; lanes 6–8: fractions from SEC; and lane 9: molecular mass standards. P-GceC and GceC are indicated by arrows. **(B)** 14-3-3 and Ftz-F1 LBD purification. SDS-PAGE analysis of 14-3-3 (lanes 1–5) and Ftz-F1 LBD (lanes 6–10) samples. Lane 1: all bacterial protein fractions; lane 2: soluble protein fraction; lane 3: fraction of proteins not bound to the Ni^2+^-NTA resin; lane 4: combined elution fractions; lane 5: 14-3-3 purified with SEC; lane 6: all bacterial protein fractions; lane 7: the soluble protein fraction; lane 8: the fraction of proteins not bound to the Ni^2+^-NTA resin; lane 9: combined elution fractions; lane 10: digested Ftz-F1 LBD purified with SEC; and lane 11: molecular mass standards. 14-3-3 and the Ftz-F1 LBD are indicated by arrows.

The 14-3-3 protein interacts with its partners by binding phosphorylated S residues (Rajagopalan et al., 2008) (although binding of non-phosphorylated proteins is also possible (Masters et al. (1999)). Therefore, samples of GceC in the phosphorylated form (P-GceC) were prepared. Its purification procedure was identical to that developed for non-phosphorylated GceC. During SDS-PAGE analysis, in the P-GceC sample, two bands were observed (Figure 2A). The upper band, with decreased mobility, refers to a phosphorylated protein (P-GceC), while the lower band refers to non-phosphorylated GceC. When alkaline phosphatase was added to the sample, mainly, a lower band (GceC) was observed, which confirms the phosphorylation of GceC (Supplementary Figure S2). During expression, approximately half of the protein was phosphorylated.

The Ftz-F1 LBD purification procedure was described previously (Kolonko et al., 2020). In short, the 6 × His -FTZ-F1 LBD fusion protein was highly overexpressed and fully soluble. First, it was bound to the Ni^2+^-NTA resin, eluted with imidazole, and digested with thrombin overnight. Finally, it was purified from digesting enzymes and proteins co-purified on IMAC by SEC on the Superdex 75 10/300 GL column. The MM of LBD Ftz-F1 was calculated based on its aa sequence (28 kDa), and its apparent mass determinate from electrophoretic mobility overlapped (Figure 2B). The Ftz-F1 LBD is a globular domain.

The 14-3-3 human isoform ζ expressed from pET-M11 was fully soluble and highly overexpressed. IMAC on the Ni^2+^-NTA resin was used as the first step of purification. After elution, 14-3-3 was concentrated and purified by SEC on the Superdex 200 10/300 GL column, separating small impurities and changing the buffer. The apparent mass determined by SDS-PAGE (Figure 2B) is in agreement with the calculated MM (30 kDa when the tag is included) since 14-3-3 is a globular protein (Sluchanko and Gusev, 2012).

The native, active form of all purified proteins was controlled by determining their ability to interact (see the following section).

### NMR analysis

To determine functional differences between MetC and GceC, their ability to interact with both the Ftz-F1 LBD and 14-3-3 was analyzed by NMR spectroscopy. To determine aa residues directly involved in the interaction, spectrum assignment, and chemical shift perturbation analysis were performed. The MetC chemical shifts were deposited in the BMRB (Hoch et al., 2023) under the accession number 51720. The MetC resonance assignment percentage was 87.5%. We performed secondary structure prediction (neighbor-corrected Structural Propensity Calculator (ncSPC)) (Tamiola and Mulder, 2012)), which clearly showed the highly unstructured nature of the protein. The highest score (up to 0.3) in two regions (vicinity of residues 518 and 639 of full Met) indicated a slight propensity toward α-helix formation in these regions. Other parts remain mostly unstructured.

### MetC assignment and interaction analysis

The 2D^15^N-HSQC spectrum of MetC, given in Supplementary Figure S3, shows features typical for IDPs, for which chemical environments of all the observed nuclei are averaged due to fast conformational changes, leading to a very narrow chemical shift range (Zuiderweg, 2002; Grudziąż et al., 2018); in the MetC case, it is 8–8.6 ppm in the proton dimension. Therefore, observed signals correlating amide proton and amide nitrogen frequencies strongly overlap. More dispersed signals (like 556E, 574E, 655V, and 716R) may correspond to aa residues involved in the formation of local and transient motifs of the secondary structure (like MoREs).

**FIGURE 3:**
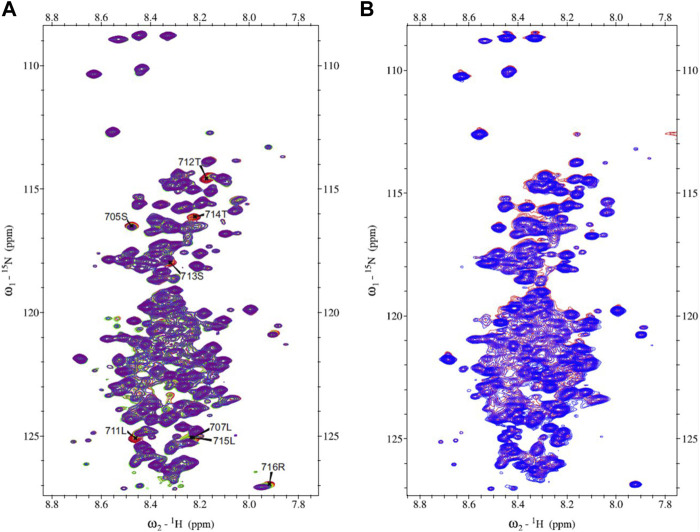
MetC chemical shift perturbation NMR spectra. **(A)** MetC NMR reference spectrum (red) and MetC spectrum obtained in the absence and presence of Ftz-F1 LBD in increasing concentrations (orange–green–blue–purple). Specific aa shifts are observed. MetC aa assignment is added. **(B)** MetC NMR reference spectrum (red) and MetC spectrum recorded in the absence and presence of an equimolar concentration of 14-3-3 (blue). No aa shifts are observed. For MetC NMR analysis, 20 mM Bis-Tris pH 6.5, 150 mM NaCl, and 5 mM βME were used as the buffer.

The ^15^N-HSQC spectrum of MetC was used for the chemical shift perturbation experiment to define interactions between MetC and the Ftz-F1 LBD and determine aa residues affected by binding. The spectra were recorded in the absence and presence of an unlabeled Ftz-F1 LBD in increasing concentrations. Multiple signals show significant changes in intensity or are shifted (Figure 3A), which unequivocally indicates interactions. The greatest chemical shift changes were observed for residues 705S, 707L, 711L, 712T, 713S, 714T, 715L, and 716R (Figure 3A).

Analogous measurements were performed to analyze interactions between MetC and 14-3-3. In accordance with *in silico* analysis (Supplementary Table S1) (Greb-Markiewicz et al., 2015), in contrast to GceC, no specific shifts were observed (Figure 3B), which indicates no interactions. The result is consistent with the hypothesis based on our previous research (Greb-Markiewicz et al., 2015) and *in silico* analysis that Gce interaction with 14-3-3 depends on the GceC presenting 14-3-3-binding motifs.

### GceC interaction analysis

The efficiency of GceC expression and purification was very low; therefore, it was not possible to obtain sufficiently concentrated samples to assign the observed signals to aa residues in the protein chain. However, samples suitable for 2D^15^N-HSQC measurements were prepared to verify if any changes in the reference spectrum in the presence of an equimolar amount of Ftz-F1 LBD or 14-3-3 can be observed.

The 2D^15^N-HSQC GceC spectrum, a characteristic of IDPs, was described previously (Kolonko et al., 2020). Specific signal shift perturbation in the presence of Ftz-F1 LBD was also presented (Kolonko et al., 2020). Here, we analyzed the ^15^N-labeled P-GceC spectrum in the presence of unlabeled 14-3-3 protein (Supplementary Figure S4). The phosphorylated form of GceC was used since 14-3-3 interacts mainly with phosphorylated S (Rajagopalan et al., 2008). The comparison of P-GceC spectra recorded in the absence and presence of 14-3-3 shows a change in intensity and shifting of many signals, which indicate an interaction between proteins. Importantly, the scheme of changes in the case of an interaction with the Ftz-F1 LBD and 14-3-3 differs: the shift perturbation depends on the partner being bound. Moreover, in the case of the interaction with the Ftz-F1 LBD, a slightly larger number of changes in the GceC spectrum was recorded (Kolonko et al., 2020), which may suggest a more robust and more stable interaction.

### Ftz-F1 assignment and interaction analysis

The interactions between full-length Ftz-F1 and Met/Gce were studied previously (Bernardo and Dubrovsky, 2012a). Here, we focused on the binding capacity of the Ftz-F1 LBD, comprising the AF2 motif, and MetC/GceC regions, consisting of the novel NR-box. In addition, we analyzed interactions of the Ftz-F1 LBD with Met^PEP^ and Gce^PEP^, which were hypothetically directly involved and responsible for interactions with Ftz-F1 (Bernardo and Dubrovsky, 2012a).

The Ftz-F1 LBD 2D^15^N-HSQC spectrum was previously presented by Daffern et al. (2019). We recorded a very similar reference spectrum pattern; however, many unsystematic shifts of the resonance positions were observed, which made it impossible to transfer the assignment directly. To support the assignment transfer, a set of 3D NMR experiments was conducted, and the observed signals were reassigned to aa residues in the Ftz-F1 LBD chain. The Ftz-F1 LBD spectrum shows better peak dispersion characteristics compared to the spectra of IDPs; however, a large number of aa residues causes a substantial level of peak overlap in the middle part of the spectrum, even for this globular protein.

**FIGURE 4:**
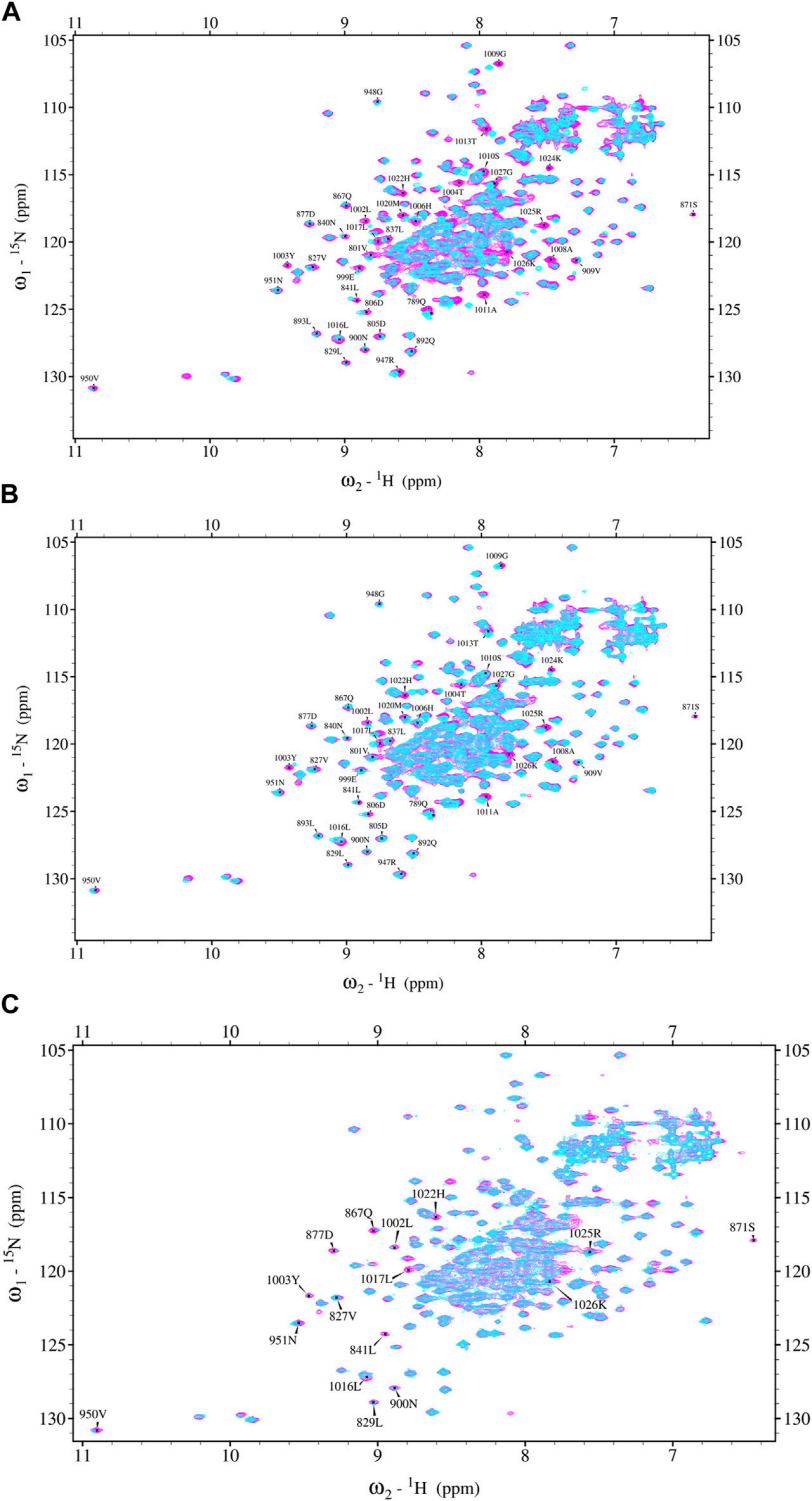
Ftz-F1 LBD chemical shift perturbation NMR spectrum. **(A)** Ftz-F1 NMR reference spectrum (magenta) and Ftz-F1 spectrum obtained in the absence and presence of MetC^PEP^ (blue). The concentration of Ftz-F1 was 450 μM, and that of the peptide was approximately 3 mM. Shifted aa residues are indicated. **(B)** Ftz-F1 NMR reference spectrum (magenta) and Ftz-F1 spectrum recorded in the presence of GceC^PEP^ (blue). The concentration of Ftz-F1 was 450 μM, and that of the peptide was approximately 2 mM. Shifted aa residues are indicated. **(C)** Ftz-F1 NMR reference spectrum (magenta) and Ftz-F1 spectrum obtained in the presence of MetC (blue). The concentration of Ftz-F1 was 450 μM, and that of MetC was 450 μM. Shifted aa residues are indicated. The buffer used for Ftz-F1 NMR analysis was 20 mM sodium phosphate pH 6.8, 50 mM NaCl, 1 mM TCEP, and 0.5 mM EDTA.

First, Ftz-F1 interaction capacity with Met^PEP^ and Gce^PEP^ was analyzed. In the presence of equimolar or double equimolar amounts of peptides, only subtle changes compared to the reference spectrum were observed. It indicated that the interaction of Ftz-F1 with both peptides is rather weak. Therefore, each peptide was added to the Ftz-F1 LBD sample up to its solubility limit (approximately 3 mM for Met^PEP^ and 2 mM for Gce^PEP^). The recorded spectra present specific signal shifts (Figures 4A, B) that confirm peptides binding to the Ftz-F1 LBD. Importantly, the scheme of observed changes is similar for each Met^PEP^ and Gce^PEP^ (the same residues were affected, but the intensity of the effect is more pronounced on the former) and ensures that peptide sequences contain Ftz-F1-binding sites and are sufficient to form a stable interaction interface with the FTZ-F1 LBD *in vitro*. The solubility of Gce^PEP^ was decreased compared to that of Met^PEP^, and thus, the observed shifts were smaller. Similar analyses were performed for the labeled Ftz-F1 LBD in the presence of equimolar amounts of MetC or GceC. Again, with the peptide-like pattern, many shift perturbations were noticed (Figure 4C, MetC). Importantly, in the case of MetC or GceC, the equimolar concertation was sufficient to observe specific shifts.

The aa residues of Ftz-F1 experiencing a major shift were 1002L, 1003Y, 1004T, 1008A, 1009G, 1010S, 1011A, 1013T, 1024K, 1025R, and 1026K (localized in the Ftz-F1 LBD helix α12, AF2 sequence). Slightly smaller shifts were observed for 1006H, 1016L, 1017L, 1020M, and 1027G (also localized in the Ftz-F1 LBD helix α12, AF2 sequence). Additional changes are observed for 947R–948G and 801V+ (referring to the α3 helix and sequence connecting α8 and α9).

### ITC titration of Ftz-F1 LBD with Met^PEP^

Isothermal titration calorimetry was used to confirm the binding of the novel NR-box represented by Met^PEP^ and Gce^PEP^ to the Ftz-F1 LBD and to determine the binding affinity. As shown in Supplementary Figure S5, the binding of Met^PEP^ to the Ftz-F1 LBD is represented as a decreasing exothermic process. The global data fitting to the 1:1 independent model uncovered micromolar affinity with a *K*_d_ value of 9.9 ± 0.5 µM and Δ*H* ^ITC^ of −1.1 ± 0.5 kcal ⋅ mol^-1^. The calculated Δ*G* ^ITC^ and Δ*S* ^ITC^ values based on the fitted ones were −6.8 kcal⋅mol^-1^ and 19.1 cal⋅mol^-1^⋅K^−1^, respectively (Padjasek et al., 2020). Since the solubility of Gce^PEP^ was very low, it was impossible to determine the *K*_d_ for this interaction due to experimental requirements.

### Pull-down analysis

To confirm the results of NMR studies and test interactions between full-length Met and Gce with partners, we performed the immunoprecipitation experiment using the ANTI-FLAG M2 Affinity Gel (Sigma-Aldrich) and COS-7 cells. The proteins were expressed as results of cell transfection with a single vector encoding CFP-Ftz-F1/LBD, CFP-14-3-3, YFP-Met-FLAG, YFP-Gce-FLAG, YFP-MetC-FLAG, or YFP-GceC-FLAG, or with two selected vectors (YFP-Met-FLAG and CFP-Ftz-F1 LBD or CFP-14-3-3; YFP-Gce-FLAG and CFP-Ftz-F1 LBD or CFP-14-3-3; YFP-MetC-FLAG and CFP-Ftz-F1 LBD or CFP-14-3-3; and YFP-GceC-FLAG and CFP-14-3-3) (YFP-GceC-FLAG in combination with the CFP-Ftz-F1 LBD was tested previously (Kolonko et al., 2020)), and pulled down from the cell lysates using the ANTI-FLAG M2 Affinity Gel. Western blotting was used to visualize the results. Importantly, a mix of proteases and inhibitors was used during cell lysis and precipitation experiments to prevent the loss of phosphate groups indispensable for the 14-3-3 interaction. CFP-Ftz-F1 LBD and CFP-14-3-3 were not tagged with the FLAG peptide and, therefore, could not bind to the ANTI-FLAG M2 Affinity Gel, providing a negative control—no band is visible in the elution fractions (Supplementary Figures S6A, B). Each YFP-Met-FLAG, YFP-Gce-FLAG, YFP-MetC-FLAG, and YFP-GceC-FLAG expressed alone was used as positive controls. The presence of the FLAG-tag at their C-termini allowed them to bind to the affinity gel. Each protein is observed as a single band at the expected area in the elution fractions (Supplementary Figures S6C, D). The additional bands observed in the elution fraction for MetC and GceC are nonspecific.

**FIGURE 5:**
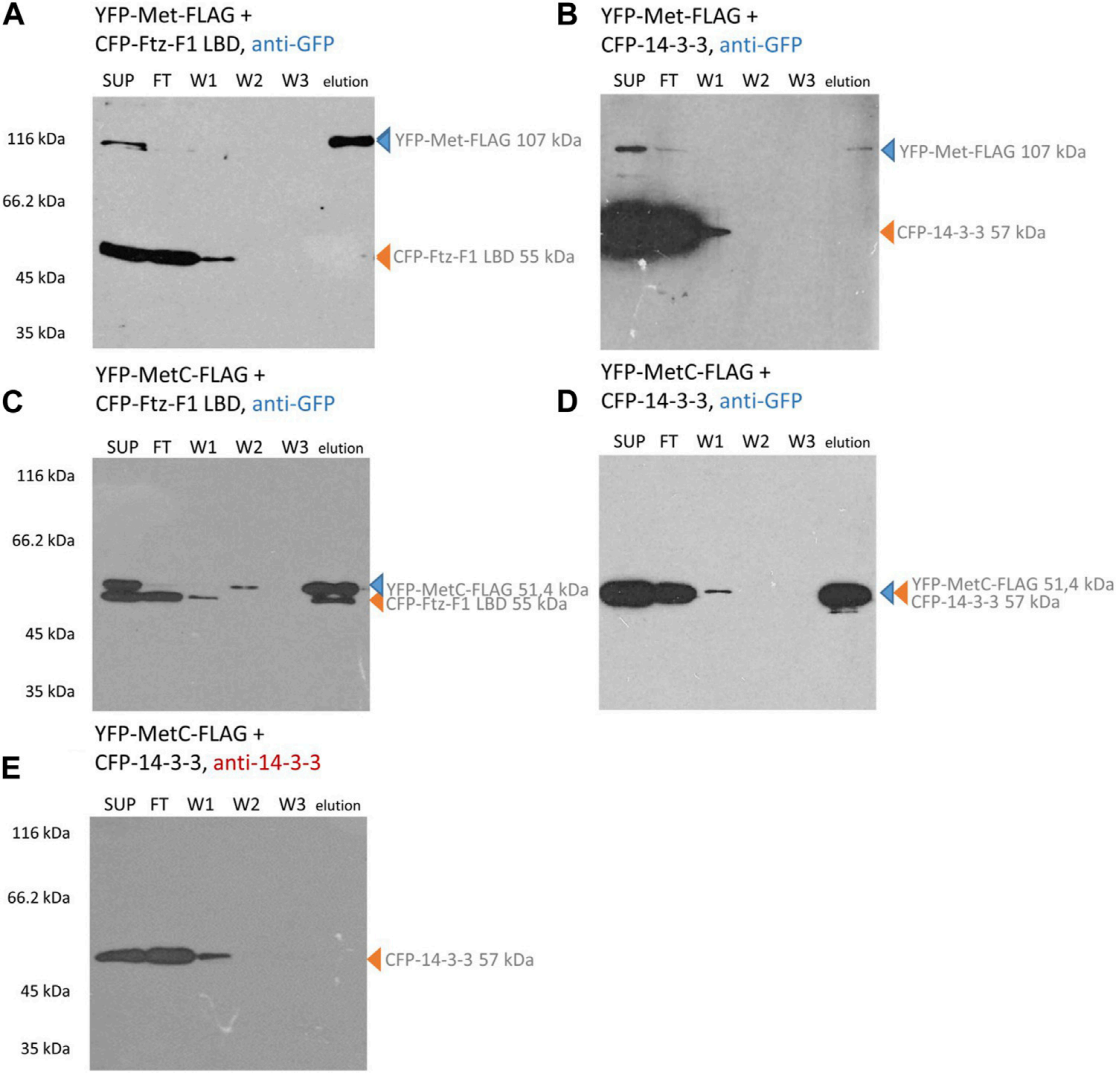
Met and MetC interaction analysis. COS-7 cells were simultaneously transfected with two selected vectors, and lysates were used for pull-down assays with the ANTI-FLAG M2 Affinity Gel. Finally, the samples were analyzed for the presence of YFP-Met-FLAG/YFP-MetC-FLAG and CFP-FTZ-F1 LBD/CFP-14-3-3 by Western blotting and detected by anti-GFP or anti-14-3-3 antibodies. In the case of YFP-Met-FLAG interaction studies **(A, B)**, the protein was present in SUP, partially in FT, and in elution fractions. The CFP-Ftz-F1 LBD (same as CFP-14-3-3) was present in SUP, FT, and W1 fractions. No bands corresponding to the CFP-Ftz-F1 LBD (or CFP-14-3-3) were present in elution fractions. No interactions were detected. In the case of YFP-MetC-FLAG **(C–E)** interaction studies, the protein is present in SUP, partially in FT, and in elution fractions. The CFP-Ftz-F1 LBD was present in SUP, FT, W1, and elution fractions. CFP-14-3-3 was present in SUP, FT, and W1 fractions. The interaction between YFP-MetC-FLAG and CFP-Ftz-F1 LBD was detected, while YFP-MetC-FLAG does not interact with CFP-14-3-3. SUP: supernatant; FT: proteins not bound to the resin; W1-3: wash fraction; and elution: elution fraction. Observed fusion proteins with MM are marked with arrows (blue for proteins with the FLAG tag, binding to the ANTI-FLAG M2 Affinity Gel, and orange for untagged proteins, not binding to the ANTI-FLAG M2 Affinity Gel). All experiments were repeated twice or more.

In contrast, the CFP-Ftz-F1 LBD and CFP-14-3-3 cannot bind to the resin and require interaction with the FLAG-labeled protein to be present in the eluted fraction. Hence, their detection with an anti-GFP antibody in the elution fraction clearly confirms the interaction. In the case of YFP-tagged Met-FLAG co-expressed with the CFP-Ftz-F1 LBD or CFP-14-3-3, only the band responding to YFP-Met-FLAG is detected in the elution fraction (Figures 5A, B). This result indicates that no interactions occurred. A similar result was obtained for YFP-MetC-FLAG co-expressed with CFP-14-3-3 (Figure 5D). Since the electrophoretic mobility of both proteins is highly similar, the results were confirmed by the detection of proteins with an anti-14-3-3 antibody (Figure 5E). CFP-tagged 14-3-3 is visible in the cell lysate (SUP), a fraction of proteins not bound to the resin (FT) and wash fraction (W1) but not in the fraction eluted from the resin. These results show that YFP-MetC-FLAG and CFP-14-3-3 do not interact. In the case of YFP-MetC-FLAG and CFP-Ftz-F1 LBD, two bands were observed in the elution fraction (Figure 5C), which indicate the interaction between proteins.

**FIGURE 6:**
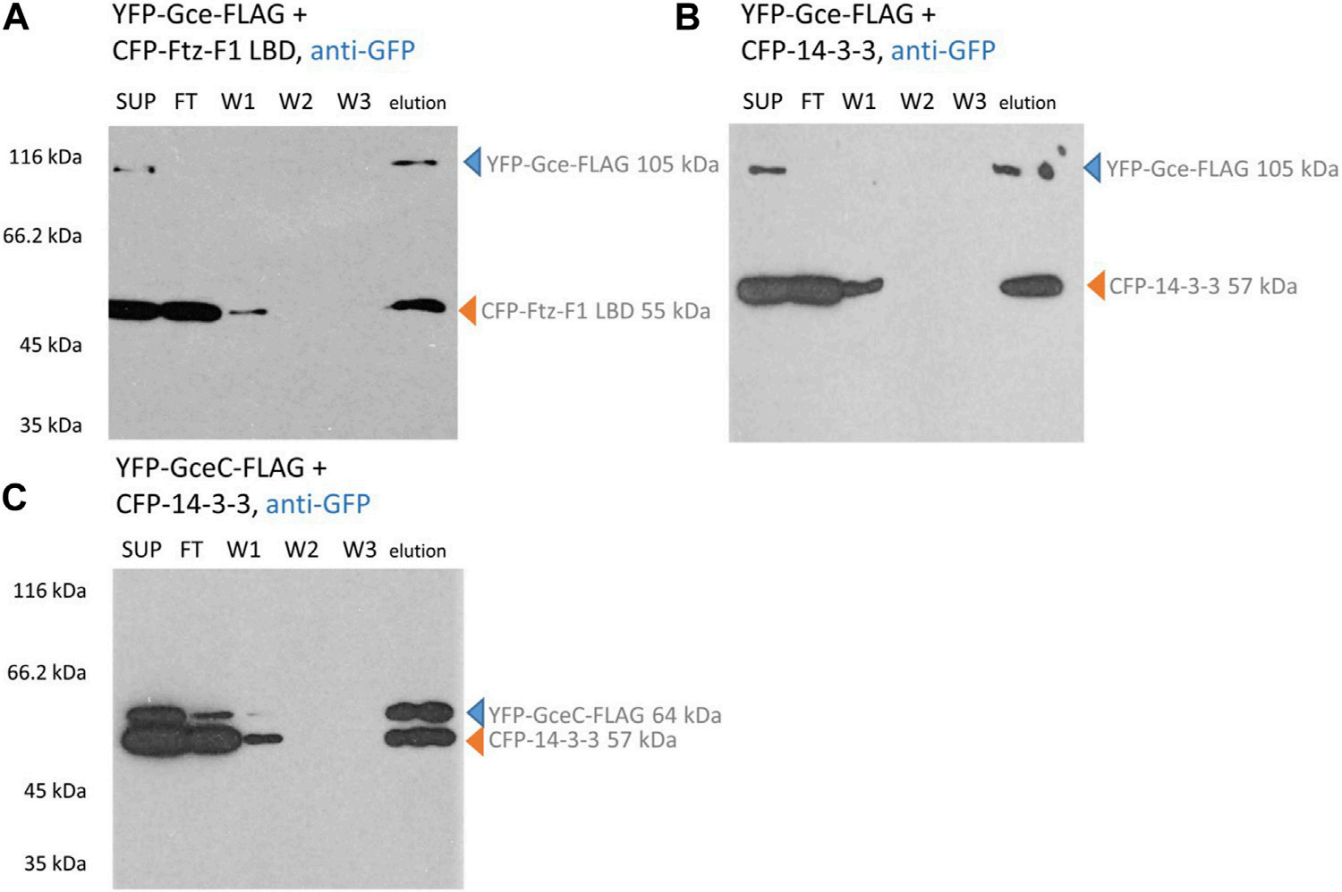
Gce and GceC interaction analysis. COS-7 cells were simultaneously transfected with two appropriate vectors and pulled down with ANTI-FLAG M2 Affinity Gel. Finally, the samples were analyzed for the presence of YFP-Gce-FLAG/YFP-GceC-FLAG and CFP-FTZ-F1 LBD/CFP-14-3-3 by Western blotting and detected by anti-GFP antibodies. **(A,B)** In the case of YFP-Gce-FLAG interaction studies, the protein was present in SUP and elution fractions. The CFP-Ftz-F1 LBD (same as CFP-14-3-3) was present in SUP, FT, partially in W1, and elution fractions. Since the CFP-Ftz-F1 LBD and CFP-14-3-3 do not have any FLAG tag, the presence of these proteins in eluted fractions is dependent on the interaction with YFP-Gce-FLAG. **(C)** In the case of YFP-GceC-FLAG interaction studies, YFP-GceC-FLAG was present in SUP, partially in FT, and elution fractions. The CFP-14-3-3 was present in SUP, FT, partially W1, and in elution fractions, which confirm interactions between YFP-GceC-FLAG and CFP-14-3-3. SUP: supernatant; FT: proteins not bound to the resin; W1-3: wash fraction; and elution: elution fraction. Observed fusion proteins with MM are marked with arrows (blue for proteins with FLAG tag, binding to the ANTI-FLAG M2 Affinity Gel, and orange for untagged proteins, not binding to the ANTI-FLAG M2 Affinity Gel). All experiments were repeated twice or more.

Similarly, YFP-Gce-FLAG co-expressed with the CFP-Ftz-F1 LBD (Figure 6A) or CFP-14-3-3 (Figure 6B) and YFP-GceC-FLAG co-expressed with CFP-14-3-3 (Figure 6C) resulted in two bands observed in the fraction eluted from the ANTI-FLAG M2 Affinity Gel. Observed bands correspond to bands expected for the tested protein pair, which clearly confirms the interactions. A similar result was observed previously for YFP-GceC-FLAG paired with CFP-Ftz-F1 LBD (Kolonko et al., 2020).

Summarizing, we showed that the full-length Gce and GceC can interact with Ftz-F1 LBD and 14-3-3. Our results show that GceC is sufficient for interactions with Ftz-F1/LBD and 14-3-3. Interestingly, the full-length Met does not interact with any of these proteins under the same conditions, and MetC can interact only with the Ftz-F1 LBD. Importantly, we showed differentiation in the binding partners of Gce and Met, which can result in different functions of these proteins. In addition, the C-termini of Gce and Met seem to be factors responsible for this differentiation.

### Analysis of the localization of interacting proteins

**FIGURE 7:**
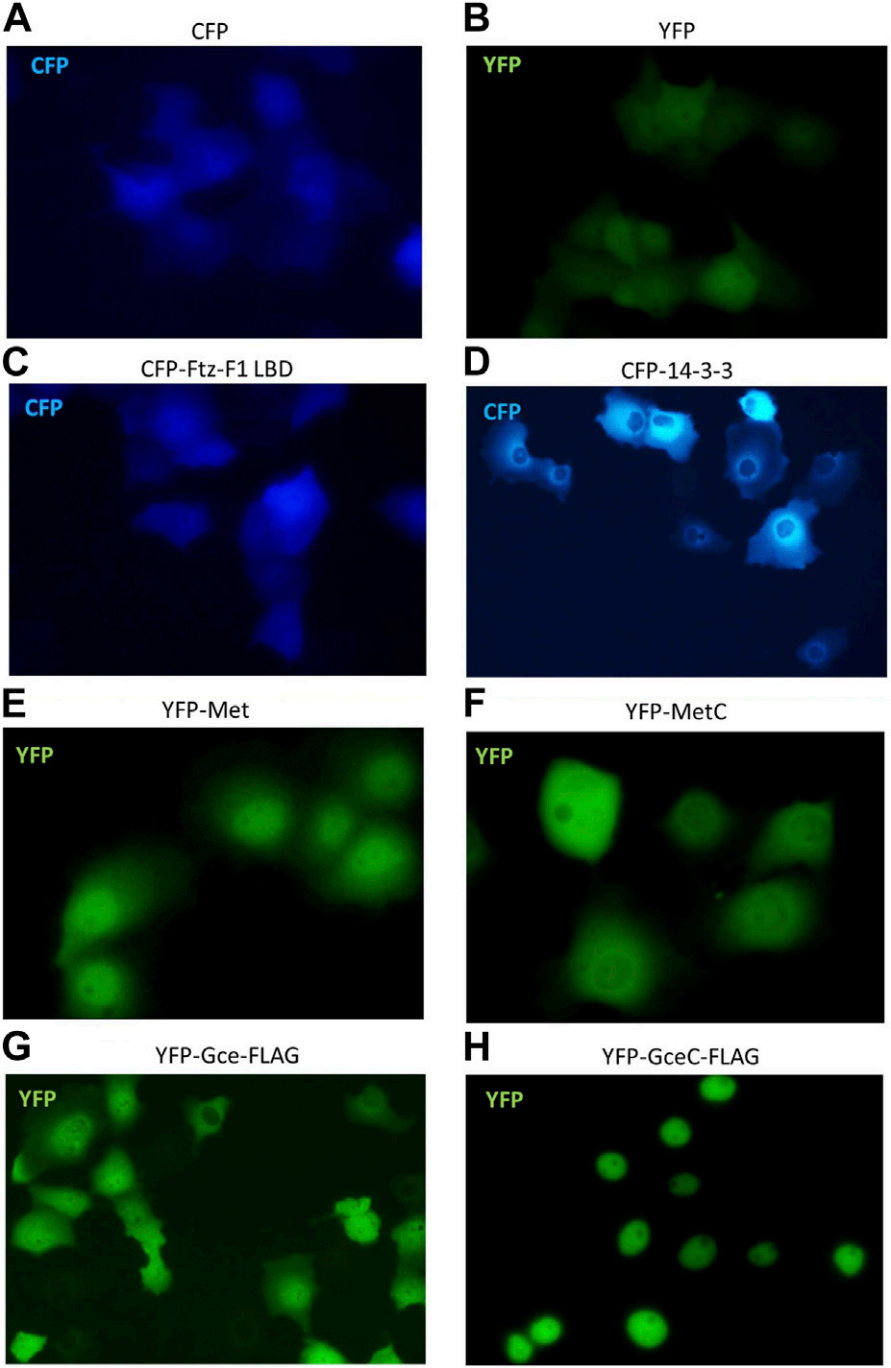
Subcellular localization of Ftz-F1 LBD, 14-3-3, Met and MetC, and Gce and GceC. The subcellular localization of proteins was analyzed 24 h after the transfection of the COS-7 cells. Representative images of the COS-7 after single transfection are presented. Cells transfected with pEYFP-C1 **(A)** or pECFP-C1 **(B)** present ubiquitous fluorescence of the YFP or CFP proteins within the cell (control). **(C)** The CFP-Ftz-F1 LBD was distributed ubiquitously within the whole cell, and **(D)** CFP-14-3-3 was present in the cytoplasm, with a substantial accumulation around the nuclei. **(E)** The YFP-Met-FLAG was distributed predominantly in the nuclei, and **(F)** YFP-MetC-FLAG was observed in the cytoplasm with some accumulation around the nuclei. **(G)** The YFP-Gce-FLAG was observed in both compartments with a slight predominance around the nuclei, while some cells (less than 5%) presented a cytoplasmic signal. **(H)** The YFP-GceC-FLAG was observed exclusively in the nucleus.

To continue interaction studies, we investigated whether interacting partners affect the subcellular localization of the tested proteins. We used fluorescence microscopy to observe the distribution of Met, MetC, Gce, and GceC in the presence and absence of the FTZ-F1 LBD and 14-3-3 in COS-7 cells and analogous to the pull-down method pEYFP-C1 and pECFP-C1 derivatives. The cells were analyzed by fluorescent microscopy 24 h after transfection of COS-7 cells. The cells transfected with a single vector were used as a control. The monitoring of protein overexpression and subcellular localization in living cells by fluorescent protein labeling is commonly used (Chalfie et al., 1994). Importantly, it was shown that GFP tagging does not affect the localization and activity of fused proteins (Chudakov et al., 2010). First, we performed a test for control proteins. As expected, cells transfected with pEYFP-C1 or pECFP-C1 presented ubiquitous fluorescence of the YFP or CFP proteins (Figures 7A, B). The expressed CFP-Ftz-F1 LBD was also distributed ubiquitously in the whole cell (Figure 7C), while CFP-14-3-3 was distributed only in the cytoplasm, with a substantial accumulation around the nuclei (Figure 7D). The expressed YFP-Met-FLAG localized predominantly in the nuclei (Figure 7E), while YFP-MetC-FLAG was in the cytoplasm with some accumulation around the nuclei (Figure 7F). Finally, the expressed YFP-Gce-FLAG fluorescence for most analyzed cells was observed in both compartments with a slight predominance of the nuclei, while some cells (less than 5%) presented a cytoplasmatic signal (Figure 7G). As expected, YFP-GceC-FLAG was observed exclusively in the nuclei (Figure 7H).

**FIGURE 8:**
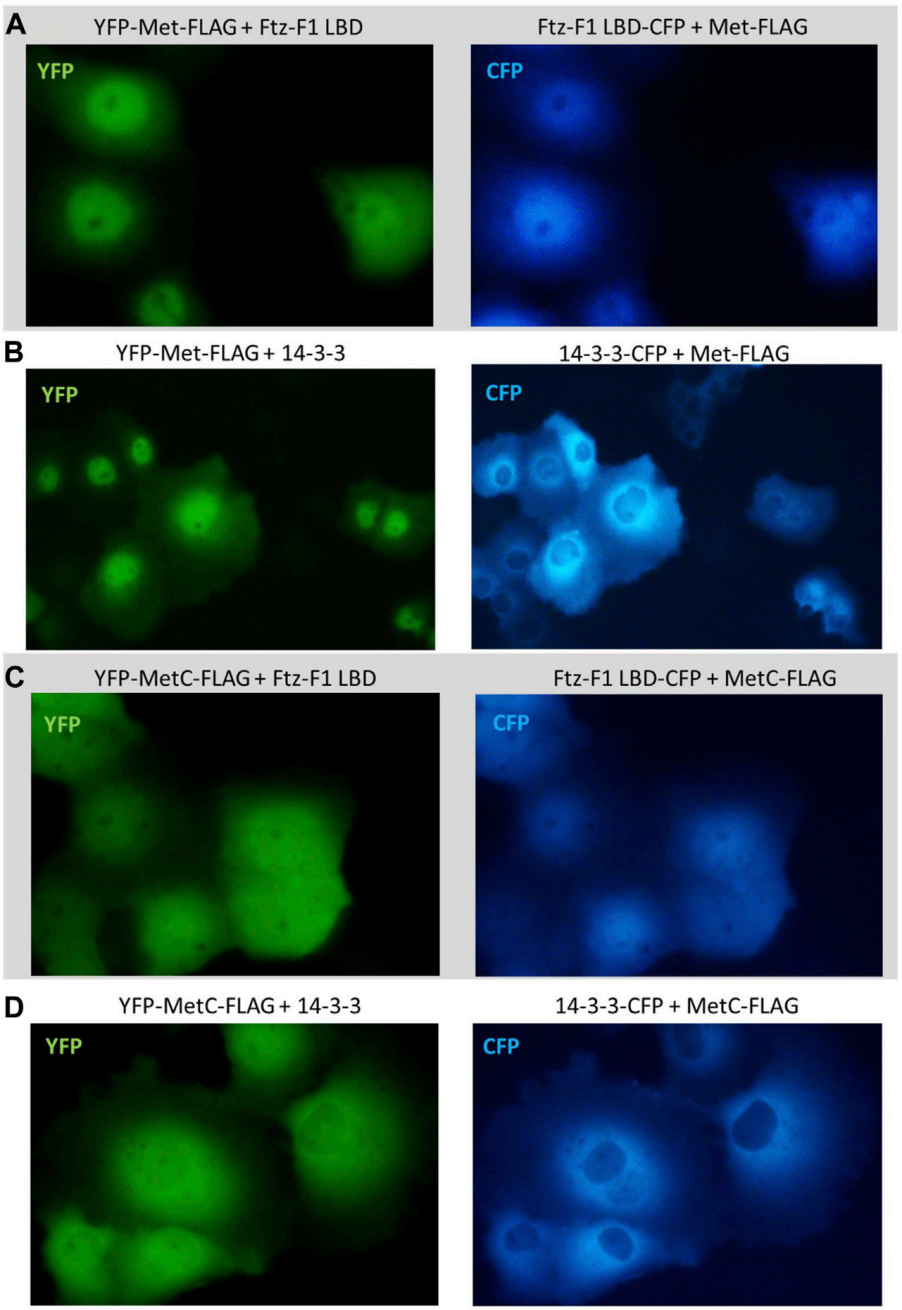
Subcellular localization of Met and MetC co-expressed with the Ftz-F1 LBD or 14-3-3. The subcellular distribution of each protein was analyzed 24 h after the transfection of the COS-7 cells. Representative images of the COS-7 after co-transfection are presented. No change in the subcellular localization pattern was observed for **(A)** YFP-Met-FLAG co-expressed with the CFP-Ftz-F1 LBD and **(B)** YFP-Met-FLAG co-expressed with CFP-14-3-3. **(C)** Both YFP-MetC-FLAG and CFP-Ftz-F1 partially shifted to the nucleus, resulting in more homogenous distribution in the analyzed cells when co-expressed. **(D)** YFP-MetC-FLAG co-expressed with CFP-14-3-3 did not present any change in the subcellular localization.

No shift was observed in the subcellular localization when YFP-Met-FLAG was co-expressed with the CFP-Ftz-F1 LBD (Figure 8A) or CFP-14-3-3 (Figure 8B) and when YFP-MetC-FLAG was co-expressed with CFP-14-3-3 (Figure 8D). In contrast, YFP-MetC-FLAG was co-expressed with the CFP-Ftz-F1 LBD, resulting in the shift of YFP-MetC-FLAG to nuclei, which, in turn, resulted in the almost homogenous distribution in both compartments of the cell, analogous to the distribution of the CFP-Ftz-F1 LBD (Figure 8C).

**FIGURE 9:**
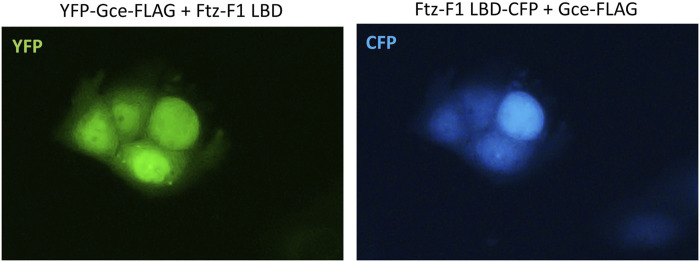
Subcellular localization of Gce co-expressed with the Ftz-F1 LBD. The subcellular distribution of each protein was analyzed 24 h after the transfection of the COS-7 cells. Representative images of the COS-7 after co-transfection are presented. Both YFP-Gce-FLAG and the CFP-Ftz-F1 LBD shifted toward the nucleus in analyzed cells.

The co-expression of YFP-Gce-FLAG with the CFP-Ftz-F1 LBD shifted both proteins toward the nucleus (Figure 9). Similar results were observed previously for YFP-GceC-FLAG in the presence of the CFP-Ftz-F1 LBD. The CFP-Ftz-F1 LBD protein was shifted to the nucleus, where YFP-GceC-FLAG was present in the absence and presence of a partner (Kolonko et al., 2020).

**FIGURE 10:**
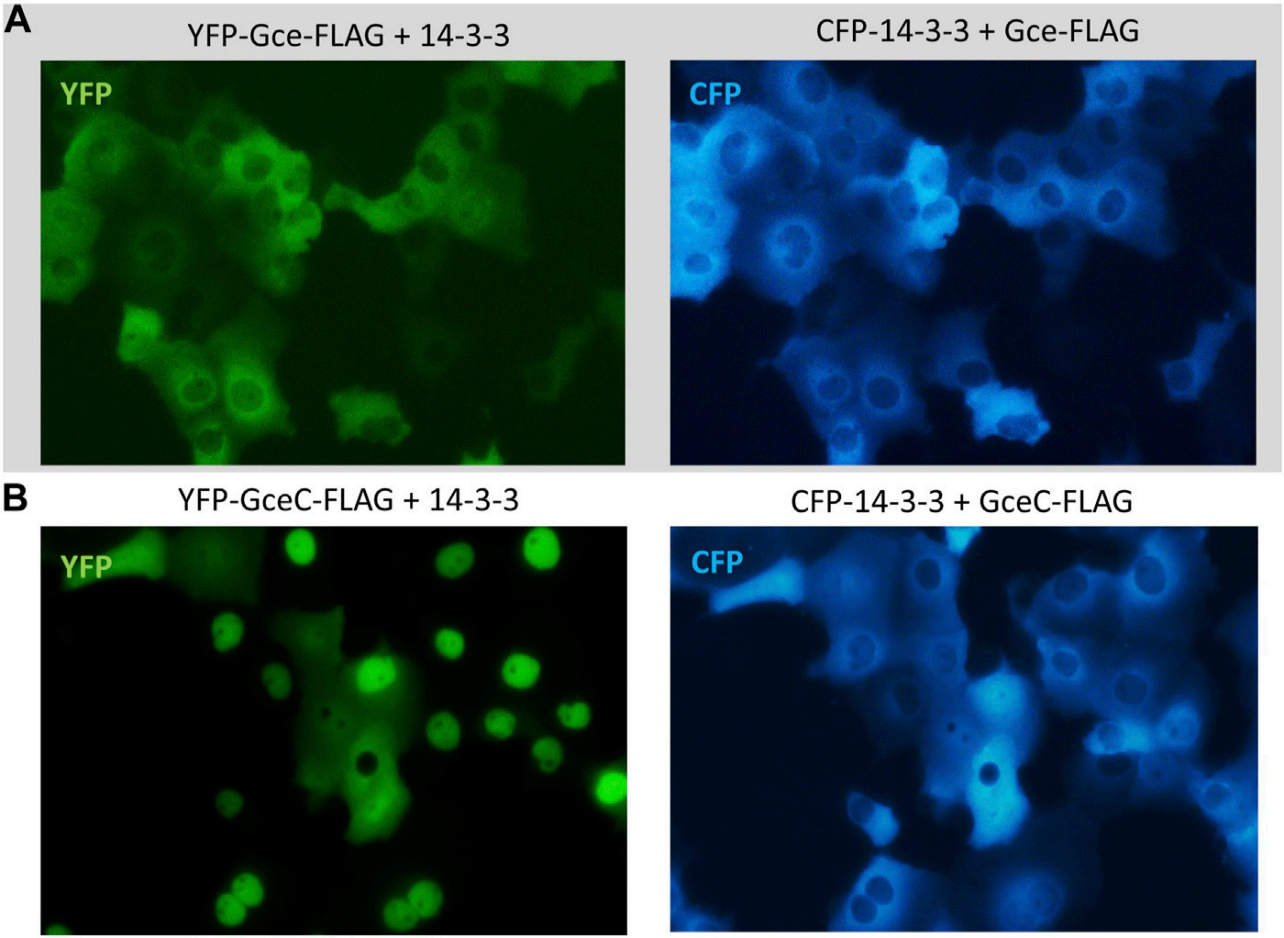
Subcellular localization of Gce and GceC co-expressed with 14-3-3. The subcellular distribution of each protein was analyzed 24 h after the transfection of the COS-7 cells. Representative images of the COS-7 after co-transfection are presented. **(A)** YFP-Gce-FLAG shifted to the cytoplasm and co-localized with CFP-14-3-3 in all analyzed cells; **(B)** YFP-GceC-FLAG transferred from the nucleus to the cytoplasm, while 14-3-3 shifted toward the nucleus in some of the analyzed cells.

Finally, we tested the impact of CFP-14-3-3 expression on the YFP-Gce-FLAG and YFP-GceC-FLAG subcellular localization. When co-expressed with cytoplasmic CFP-14-3-3, YFP-Gce-FLAG was also located in the cytoplasm (Figure 10A). In the case of YFP-GceC-FLAG, the expressed protein distribution was not ubiquitous. For approximately 50% of the analyzed cells co-transfected successfully, we observed the shift of the fluorescent signal from the nuclei to the cytoplasm (Figure 10B). Simultaneously, CFP-14-3-3 shifted toward the nuclei (Figure 10B).

We showed that a shift in the subcellular distribution of one or both partner proteins was observed for cells expressing Gce/GceC in the presence of FTZ-F1 LBD/14-3-3. In the case of MetC co-expressed with the FTZ-F1 LBD, we also observed the transfer in contrast to co-expression with 14-3-3, while the full-length Met did not present any translocation. Our results confirm that interacting partners affect the subcellular localization of the tested proteins and are consistent with results of pull-down experiments showing that full-length Met does not bind to the FTZ-F1 LBD or 14-3-3, while MetC can interact only with the FTZ-F1-LBD. The presented results allow us to document functional differences in Gce/GceC and Met/MetC behavior.

## Discussion

Met and Gce were shown as JH receptors regulating the development of *D. melanogaster*; however, their functions are not fully redundant, and each protein presents tissue specificity. Met and Gce belong to the bHLH-PAS TF family. The similarity in the sequences of bHLH-PAS family members is limited to the defined domains (bHLH and PAS), while their long C-termini are significantly variable and present characteristics of intrinsically disordered regions (IDRs) (Kolonko et al., 2016; Kolonko et al., 2020). C-terminal localization of highly flexible fragments is frequently observed in proteins (Baumann, et al., 2010b), and for bHLH-PAS factors, C-termini perform specific regulatory functions determining protein activity (Furness, et al., 2007; Uversky, 2013). For example, the mammalian Sim protein exists in two isoforms, Sim1 and Sim2, which differ in C-terminal sequences. While Sim1 activates specific gene expression, Sim2 presents the opposite activity and inhibits expression (Moffett et al., 1997). Moreover, not well-defined and highly flexible structures allow IDRs to interact with several targets (Tompa, 2002; Oldfield et al., 2005; Uversky, 2011).

Previously, we structurally characterized Met and Gce C-terminal fragments (MetC, 509-716 aa, and GceC, 661–959 aa) as IDRs (Kolonko et al., 2016; 2020). GceC, defined as less compacted and comprising more short-ordered fragments acting as molecular recognition elements (MoREs) (Kolonko et al., 2020), is predicted to interact with more partners than MetC.

The aim of this study was to understand the origin of functional differences between Met and Gce, concerning their ability to interact with two selected partners, nuclear receptor Ftz-F1 and regulatory protein 14-3-3. To test the interactions, we performed extensive NMR spectroscopy studies supported by the pull-down assay. Met and Gce present a high IDR content, resulting in low expression efficiency and poor solubility, so the full-length proteins are difficult (if not impossible) to obtain in bacterial cell culture (Kolonko and Greb-Markiewicz, 2019). Therefore, to perform the interaction analysis *in vitro*, we focused on protein regions that comprise Ftz-F1 LBD- and/or 14-3-3-binding sites: MetC and GceC (and P-GceC for interactions tests with 14-3-3).

NMR studies allowed for MetC resonance assignment (Supplementary Figure S3). It was not trivial since MetC belongs to IDPs, but it was completed by employing high-dimensional techniques providing sufficient peak resolution. The MetC shift perturbation NMR experiment clearly confirmed interactions between MetC and the Ftz-F1 LBD. The dissociation constant determined by ITC for Ftz-F1 and Met^PEP^ was 9.9 µM, and the data showed a 1:1 binding stoichiometry ratio. A similar result (15.2 µM) was obtained for the Ftz-F1 LBD titrated with Ftz^PEP^, representing a canonical NR-box (LXXLL) (Yoo et al., 2011; Daffern et al., 2019), which confirms that Met is a physiological Ftz-F1 coactivator. As shown previously (Kolonko et al., 2020), the Gce C-terminal region can also interact with the Ftz-F1 LBD.

The second part of the NMR experiments is based on the Ftz-F1 LBD shift perturbation. First, partial Ftz-F1 LBD assignment was performed to determine aa residues most affected upon binding. Comparison of the Ftz-F1 LBD reference spectrum with the spectra obtained in the presence of MetC and GceC (Kolonko et al., 2020) or Met^PEP^ and Gce^PEP^ confirmed interactions and indicated that even short peptides are sufficient to force significant structural changes in the Ftz-F1 LBD. Moreover, signal shifts observed for each MetC, GceC, Met^PEP^, and Gce^PEP^ overlap, confirming that the novel NR-box is a sequence directly interacting with Ftz-F1. Importantly, MetC/GceC binds to the Ftz-F1 LBD with higher affinity since observed shift perturbations are strong even if the concentration of the used JH receptors is low. Compared to studied peptides, using the full MetC/GceC fragment allows the creation of an enriched interaction pattern. The most perturbing aa residues of the Ftz-F1 LBD are located between 1,002 and 1,027 aa. This region comprises an evolutionarily conserved AF2 motif (1021-PTQTLLMEMLHAKRK-1026) (Schwartz et al., 2001) critical for Ftz-F1 *in vivo* activity. AF2, spanning the Ftz-F1 LBD C-terminal helix 12 (Yoo et al., 2011), is highly mobile in the aporeceptor. The coactivator binding induces AF2 conformational changes, enabling ligands and partners to recognize the surface comprising AF2 and helixes α3–α4 (Savkur and Burris, 2004; Daffern et al., 2019). The Ftz-F1 LBD spectrum measured in the presence of the canonical Ftz^PEP^ coactivator presents a similar shift pattern (Daffern et al., 2019) to that presented here; however, additional changes around 861–867 aa residues referring to helix α4 are observed (Daffern et al., 2019). It makes the interaction more stable and robust since the canonical coactivator, in addition to hydrophobic interactions with AF2, also creates electrostatic bonding with adjacent helices (Daffern et al., 2019).

Previously (Greb-Markiewicz et al., 2015), we suggested the 14-3-3 regulatory protein as a Gce partner, regulating its subcellular localization (Greb-Markiewicz et al., 2015). S732 (corresponding to S462A in the study by Greb-Markiewicz et al. (2015)) and S940 (corresponding to S670A in the study by Greb-Markiewicz et al. (2015)) were precisely predicted with the highest scores to be phosphorylated and to be a part of the 14-3-3 protein-binding motifs of Gce (Scansite (Obenauer et al., 2003), Supplementary Table S1) (Greb-Markiewicz et al., 2015). As we showed, GceC S732A and/or S940A mutants, mimicking the lack of 14-3-3-binding sites, were localized in the cytoplasm of COS-7 cells in the absence of JH and transferred to the nucleus after JH addition (Greb-Markiewicz et al., 2015). It is worth mentioning that recently, Gao et al. (2022) claimed the lack of positive prediction for 14-3-3-binding sites in Met and Gce. However, they did not reference the used programs/servers. Therefore, we cannot comment on this and rely on the results of our analysis based on the predictions of Scansite 2.0 (Obenauer et al., 2003), ELM (Dinkel et al., 2012), and 14-3-3Pred (Madeira et al., 2015), indicating interaction sites in Gce but not Met in the sequences (ELM and 14-3-3Pred results are presented and discussed in the study by Greb-Markiewicz et al. (2015)).

Since 14-3-3 proteins, highly conserved across different organisms, are expressed in all eukaryotic cells, we proposed previously that 14-3-3 present in mammalian COS-7 cells used in the study might interact with Gce, affecting protein localization and, thus, activity. Therefore, in this study, we decided to analyze Met/Gce interactions with the mammalian 14-3-3 protein, specifically with human 14-3-3 human isoform ζ as it is highly homological to the *D. melanogaster* 14-3-3 ζ isoform (92% identity determined using BLAST (Altschul et al., 1990)).

The 14-3-3 proteins usually recognize classical RSXpSXP, RX(Y/F)XpSXP, or nonclassical motifs and interact with phosphorylated S residues (Ganguly et al., 2005; Obsilova et al., 2008; Rajagopalan et al., 2008)). Moreover, 14-3-3 typically binds as a dimer with two partner protein sites (Dougherty and Morrison, 2004). Of the two D*rosophila* JH receptors, only Gce is predicted to interact with 14-3-3 in a phosphorylation-dependent manner (potential phosphorylated interaction sites are presented directly in GceC; Supplementary Table S1) (Greb-Markiewicz et al., 2015). In fact, a more elongated GceC structure (Kolonko et al., 2020) makes it more suitable for PTMs like phosphorylation (Gao et al., 2022). Importantly, the phosphorylation patterns of both Met and Gce differ (Gao et al., 2022), and most phosphorylation sites are localized in the Met N-terminus and Gce C-terminus (Gao et al., 2022). Such differentiation can easily affect the physiological functions of Met and Gce since phosphorylation enhances the DNA-binding property of Met/Gce, which facilitates JH signaling (Ojani et al., 2016). We hypothesize that GceC, more susceptible to PTMs, can be physiologically more active receptors and act independently in the presence of JH. In fact, single JH receptor homologs occurring in other species of insects present higher sequence similarity to Gce than Met (Wang, et al., 2007). The GceC shift perturbation NMR experiment demonstrated its ability to bind directly to 14-3-3 in phosphorylated form. Notably, this is an exceptional competence of GceC, not MetC.

As shown, IDPs and IDRs usually adopt more ordered conformation when interacting with partners (Uversky, 2010). We hypothesize that each interacting partner can force disordered MetC and GceC to change the structure in a partner-dependent manner. Indeed, the GceC NMR spectra obtained in the presence of each Ftz-F1 and 14-3-3 differ. Induced conformational changes in the C-termini might be propagated over the entire length of the proteins, which influences their activity.

The obtained *in vitro* results were verified by the pull-down assays in the COS-7 cell line. Importantly, for these experiments also, the full-length Met and Gce were used. As presented, GceC and Gce interact with the Ftz-F1 LBD and 14-3-3. No interactions were observed for Met, and only MetC could bind to the Ftz-F1 LBD exclusively. Such results are consistent with those of the described NMR analysis and our previous structural analysis (Kolonko et al., 2016; Kolonko et al., 2020). GceC, presenting a highly elongated structure, is separated from the protein core (Kolonko et al., 2020), and the sequences responsible for partner binding are also easily accessible in the full-length protein. Consequently, the full-length Gce does interact with both Ftz-F1 and 14-3-3, which may result in its or its partners’ translocation, activity modification, and induction of specific gene expression in the nuclei (like the expression of the E75A nuclear receptor) (Dubrovsky et al., 2011). In turn, MetC adheres very closely to the protein core, and the binding sites localized in the C-terminus are hidden in the full-length protein (Kolonko et al., 2016; Kolonko et al., 2020). Therefore, only separated MetC can bind to Ftz-F1. However, as shown by Dubrovsky et al. (2011), the full-length Met interacts with Ftz-F1 in a JH-dependent manner*.* Conformational changes induced by JH binding can be propagated to the C-terminus and cause structural changes. The “opening” of the C-terminal domain forced by JH binding most likely allows for binding site exposure, increasing the affinity of Met for interaction partners like Ftz-F1.

In this study, we documented the direct interactions of Gce and GceC with 14-3-3 by pull-down assay in mammalian cells in the absence of JH. In contrast, Met and MetC pull-down experiments were negative. It is worth mentioning that our results are inconsistent with those reported by Gao et al. (2022) claiming that the interaction of both Gce and Met with 14-3-3 is possible only in an indirect way, through the Hsp83 chaperon protein, orthologous to vertebrate Hsp90. However, Jindra et al. (2021) reported the dissociation of the Met-Hsp83 complex after the addition of JH. Hsp83 has been shown to facilitate the methoprene-stimulated nuclear import of Met (He et al., 2014), similar to Hsp90 enabling the nuclear import of the aryl hydrocarbon receptor (Ahr) mammalian homolog of Met (Kazlauskas et al., 2001). In the absence of a ligand, Hsp90 binds to the bHLH and PAS-B domains of AHR and stabilizes it within a transcriptionally inactive cytoplasmic complex (Soshilov and Denison, 2011). Importantly, a direct interaction of bHLH-PAS TF with 14-3-3 was shown for Bmal1 (Dang et al., 2016). We hypothesize that in the absence of JH, Gce interacts with 14-3-3, which prevents its activation. The addition of JH may activate an unknown factor leading to the dissociation of the Gce-14-3-3 complex that would enable the Gce translocation toward the nucleus.

The functional differentiation of Met and Gce can be partially related to the subcellular distribution of these proteins, resulting from different patterns of NLSs and NESs (Greb-Markiewicz et al., 2011; Greb-Markiewicz et al., 2015). The activity of localization signals can be affected by the direct masking of signals or specific interactions that induce conformational changes. It may be significant that NLSs/NESs are MoREs forming local and transient motifs of the secondary structure, located usually in IDRs subjected to intensive PTMs regulating the activity of signals. For these reasons, subcellular transfer and protein localization, which are crucial for its functionality, are tightly and differentially regulated. We investigated the relationship between the ongoing partner interaction and the localization pattern of Met, MetC, Gce, and GceC. We expressed fluorescently tagged proteins analogously for co-immunoprecipitation tests. Fluorescence microscopy showed the localization shift of interacting partners. The shift of Gce and GceC to the cytoplasm resulted in co-localization with 14-3-3 when co-expressed (Figures 10A, B), confirming the role of 14-3-3 as a negative regulator of the nuclear import (Gao et al., 2022). The binding of 14-3-3 may mask the NLS located in GceC and decrease its activity, which results in the decreased translocation of Gce/GceC to the nucleus. The decrease in the JH receptor transport toward the nucleus prevents the expression of the JH primary response gene *Kruppel homolog 1* (Kr-h1) (He et al., 2014). In contrast, the Ftz-F1 LBD, co-expressed with Gce or GceC, was transferred toward nuclei (Figure 9) (Kolonko et al., 2020), which allows for specific Ftz-F1-dependent gene expression. The full-length Met does not bind to the FTZ-F1 LBD or 14-3-3, and no localization shift of proteins was observed (Figures 8A, B). MetC interacting with the co-expressed Ftz-F1 LBD presented a partial shift from the cytoplasm and localized more homogenously in both cell compartments, resembling the usual pattern of Ftz-F1 (Figure 8C). All the presented results of localization studies are consistent with those of the pull-down assay and clearly show differences between Gce/GceC and Met/MetC behavior in the presence of Ftz-F1 LBD and 14-3-3.

To date, Met and Gce are often presented as equivalent JH receptors (Abdou et al., 2011), despite reports presenting evidence of only partial homolog redundancy (Dubrovsky et al., 2011). Therefore, determined structural and functional features are erroneously transferred from one paralog to another. Here, we present differences between Met and Gce based on their interaction patterns with the two selected partners. We discuss functional differentiation in the context of the C-termini structures of the receptors. As Gce was shown to be a less compact protein more susceptible to PTMs and presenting more complicated NLS/NES patterns, it is possible that it can interact with more physiological partners, also JH independently. Therefore, Gce activity might be regulated in a much more complex manner than Met activity. Importantly, we assume that Met and Gce should be treated as two individual research objects that differ in molecular characteristics and physiological activity.

## Conclusion

This study aimed to identify differences between Met and Gce functioning based on their interaction patterns with two selected partners, Ftz-F1 and 14-3-3. As presented, GceC and Gce interact with Ftz-F1 LBD, while no interactions were observed for Met, and only MetC, in the absence of other parts of Met, could bind to Ftz-F1 LBD. The interactions with 14-3-3 are an exceptional competence of Gce (and GceC). Finally, we referred to the different Met/Gce interaction patterns in the structural data of GceC (Kolonko et al., 2020) and MetC (Kolonko et al., 2016). Intrinsically disordered C-termini are responsible for interactions with several partners, contributing to the crossing of JH and 20E signaling pathways. GceC presents a highly elongated structure in which the sequences responsible for partner binding are separated from the protein core and accessible in the full-length protein (Kolonko et al., 2020). In contrast, MetC adheres closely to the protein core, and the binding sites localized in the C-terminus are hidden in the full-length protein (Kolonko et al., 2016). We hypothesize that each interacting partner can force disordered MetC and GceC to change the structure in a partner-specific and dependent manner that determines protein activity. The presented differences between Met and Gce can be crucial for their subcellular localization and functioning during the development and adulthood of *D. melanogaster*. The higher accessibility of the Gce C-terminus in the full-length protein can explain the partial redundancy of Met/Gce functions and suggests Gce as a more active and universal paralog consistent with the theory of Gce as an ancestor gene.

We believe that the results presented here will contribute to a better understanding of the molecular basis of the functioning of bHLH-PAS receptors and will justify the need to conduct research independently for Met and Gce.

## Data Availability

The datasets presented in this study can be found in online repositories. The names of the repository/repositories and accession number(s) can be found in the article/Supplementary Material.

## Abbreviations

20E: 20-hydroxyecdysone; AF2: activation function 2; bHLH-PAS: basic helix–loop–helix/Per–Arnt–Sim; FISC: βFtz-F1-interacting steroid receptor coactivator; Ftz-F1: orphan nuclear receptor fushi tarazu factor-1; Gce: germ cell-expressed; GceC: Gce C-terminus; IMAC: immobilized metal affinity chromatography; ITC: isothermal titration calorimetry; JH: juvenile hormone; *K*_d_: dissociation constant; LBD: ligand-binding domain; Met: methoprene tolerant; MetC: Met C-terminus; MoREs: transient motifs of the secondary structure; NES: nuclear export signal; NLS: nuclear localization signal; NMR: nuclear magnetic resonance; NR-box: nuclear receptor box; PCR: polymerase chain reaction; SRC: steroid receptor coactivator; TAD: transactivation domain; TAI: steroid-hormone coactivator Taiman; and TF: transcription factor.
