# Supplementary material for: Interaction patterns of methoprene-tolerant and germ cell-expressed Drosophila JH receptors suggest significant differences in their functioning
Source: Front Mol Biosci. 2023 Aug 15;10:1215550. doi: 10.3389/fmolb.2023.1215550 (PMC10465699; doi:10.3389/fmolb.2023.1215550)
Supplement: Supplementary file 1 [file DataSheet1.pdf]

Supplemental information

Table S1. Predicted 14-3-3 binding sites in the Met and Gce sequence. The prediction was made with Scansite (Obenauer, Cantley and Yaffe, 2003). *This webserver predicts 14-3-3-binding sites by searching for 14-3-3 mode-1 binding motif recognized by HUMAN 14-3-3 Protein Zeta. The probability of binding is presented.*

	aa Residue	Probability	Sequence
**Gce**	S11	low	ASRSRNS[S]TSHSQGR
	S128	low	DLMLRPP[S]NSMYHFN
	S458	low	LARAGPR[S]EPTAYEV
	S732	medium	DDGARSM[S]EFGDPYG
	S940	medium	KASTRKS[S]DSDRNQE
**Met**	**aa Residue**	**Probability**	**Sequence**
	S32	low	SGSGSS[S]DPANGRE
	S308	low	GCFRRSD[S]SLTGGAA

Table S2. Experimental parameters of resonance assignment experiments for MetC protein.

Experiment	indir dim1			indir dim2			indir dim3			indir dim4			ni	exp time (h)
	nucl	sw (Hz)	sgs	nucl	sw (Hz)	sgs	nucl	sw (Hz)	sgs	nucl	sw (Hz)	sgs		
3D HNCO	C’	2600	130	N	2300	175	-	-	-	-	-	-	2000	12.75
5D HN(CA)CONH	H^N	5600	60	N	2300	62	C’	2600	70	N	2300	62	2000	52.5
5D (HACA)CON(CA)CONH	C’	2600	70	N	3250	85	C’	2600	70	N	2300	62	2000	53.5
5D HabCabCONH	Hab	5600	60	Cab	13000	92	C’	2500	70	N	2300	62	2000	43

Tables’ abbreviations: nucl – nucleus, indir dim – indirect dimension, sw – spectral width, sgs – sampling grid size, ni – number of non-uniform sampling complex points (randomly chosen from the sampling grid).
The MetC chemical shifts were deposited in the BMRB [65], under the accession number 51720.

Table S3. Experimental parameters of resonance assignment experiments for Ftz-F1 protein.

Experiment	indir dim1			indir dim2			ni	exp time (h)
	nucl	sw (Hz)	sgs	nucl	sw (Hz)	sgs		
3D HNCO	C’	3300	90	N	3500	96	3200	20
3D HN(CA)CO	C’	3300	90	N	3500	96	conv exp	42
3D HN(CO)CA	CA	7000	70	N	3500	96	conv exp	42.5
3D HNCA	CA	7000	70	N	3500	96	conv exp	42
3D CBCA(CO)NH	CAB	14000	98	N	3500	96	conv exp	51

Tables’ abbreviations: nucl – nucleus, indir dim – indirect dimension, sw – spectral width, sgs – sampling grid size, ni – number of non-uniform sampling complex points (randomly chosen from the sampling grid) - for non-uniform sampling experiment (HNCO only), conv exp – conventional experiment: all grid points were collected.

Table S4. Sequences of primers used in PCR

The primers used for protein cDNA amplification introduces restriction site sequences for the selected endonucleases (underlined in the primer sequences). The upper-case letters in the primer sequence represent the sequence present in the protein sequence. The reverse primers for Met, Gce, MetC, and GceC introduced the C-terminal FLAG protein sequence (DYKDDDDK, marked in blue).

Met	Forward: 5’-ccccaagcttcgATGGCAGCACCAGAGACGGGC-3’ (HindIII)<br>Reverse: 5’-cggccccgggtcacttgtcgtcatcgtctttgtagtcTCGCAGCGTGCTGGTCAG-3’ (XmaI)
Gce	Forward: 5’-ccccaagcttcgATGTTTCAGCTCTCGAATAGT-3’ (HindIII)<br>Reverse: 5’-cggccccgggtcacttgtcgtcatcgtctttgtagtcGTCCTGGTCGTCCTCCTG-3’ (XmaI)
MetC	Forward: 5’-ccccaagcttcgGCGGGCCGGCAAAAGGTG-3’ (HindIII)<br>Reverse: 5’-cggccccgggtcacttgtcgtcatcgtctttgtagtcTCGCAGCGTGCTGGTC-3’ (XmaI)
GceC	Forward: 5’-ccccaagcttcgATCAACACACAGATACCGCAG-3’ (HindIII)<br>Reverse: 5’-cggccccgggtcacttgtcgtcatcgtctttgtagtcGTCCTGGTCGTCCTCCTG-3’ (XmaI)
Ftz-F1 LBD	Forward: 5’-gcgcctcgagccATGCTGGAAGAT-3’ (XhoI)<br>Reverse: 5’-ggccggatccCTATCCCTTGCGC-3’ (BamHI)
14-3-3	Forward: 5’-gcgcctcgagccATGGATAAAAATGAGCTGGTT-3’ (XhoI)<br>Reverse: 5’-ggccggatcctcaTTAATTTCCCCTCCTTCTCCT-3’ (BamHI)

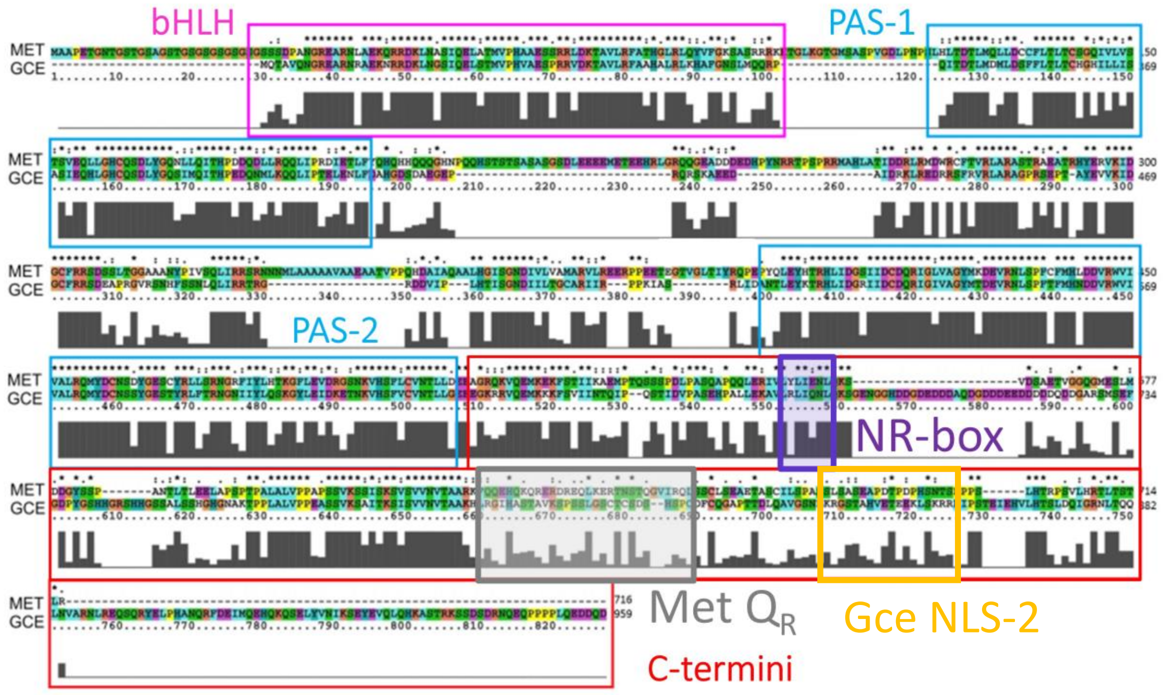

Figure S1. Met and Gce sequences alignment. *In contrast to bHLH and PAS domains (marked with pink and blue) presenting high sequence homology (78% for bHLH, 68% for PAS-1, and 86% for PAS-2) (Moore et al., 2000), C-termini of Met and Gce (MetC and GceC, marked with red) are highly differentiated.* ***.*** *Specific sequences localized in MetC and GceC are marked: NR-box with purple; Met* Q_R *sequence with grey, and Gce NLS with yellow. The alignment is based on the SIM server* (Huang and Miller, 1991).

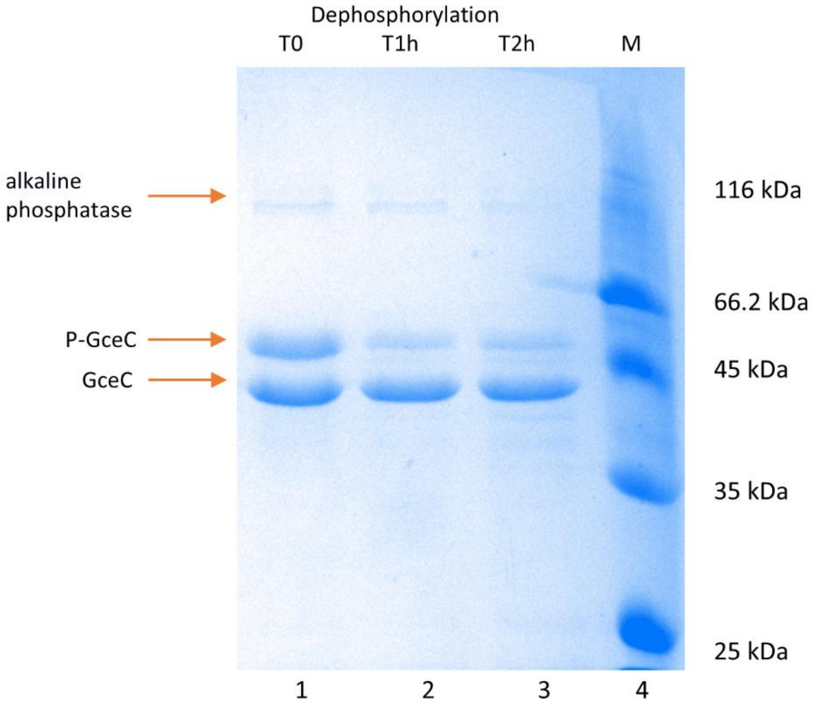

Figure S2. P-GceC dephosphorylation. *SDS-PAGE analysis of the P-GceC digested with phosphatase for 2h. Lane 1, not digested P-GceC; lane 2, protein after digestion with phosphatase for 1h; lane 3, protein after digestion with phosphatase for 2h; lane 4, molecular mass standards. The upper band, with reduced mobility, refers to a phosphorylated protein (P-GceC), while the lower band refers to non-phosphorylated GceC. Arrows indicate P-GceC, GceC and phosphatase.*

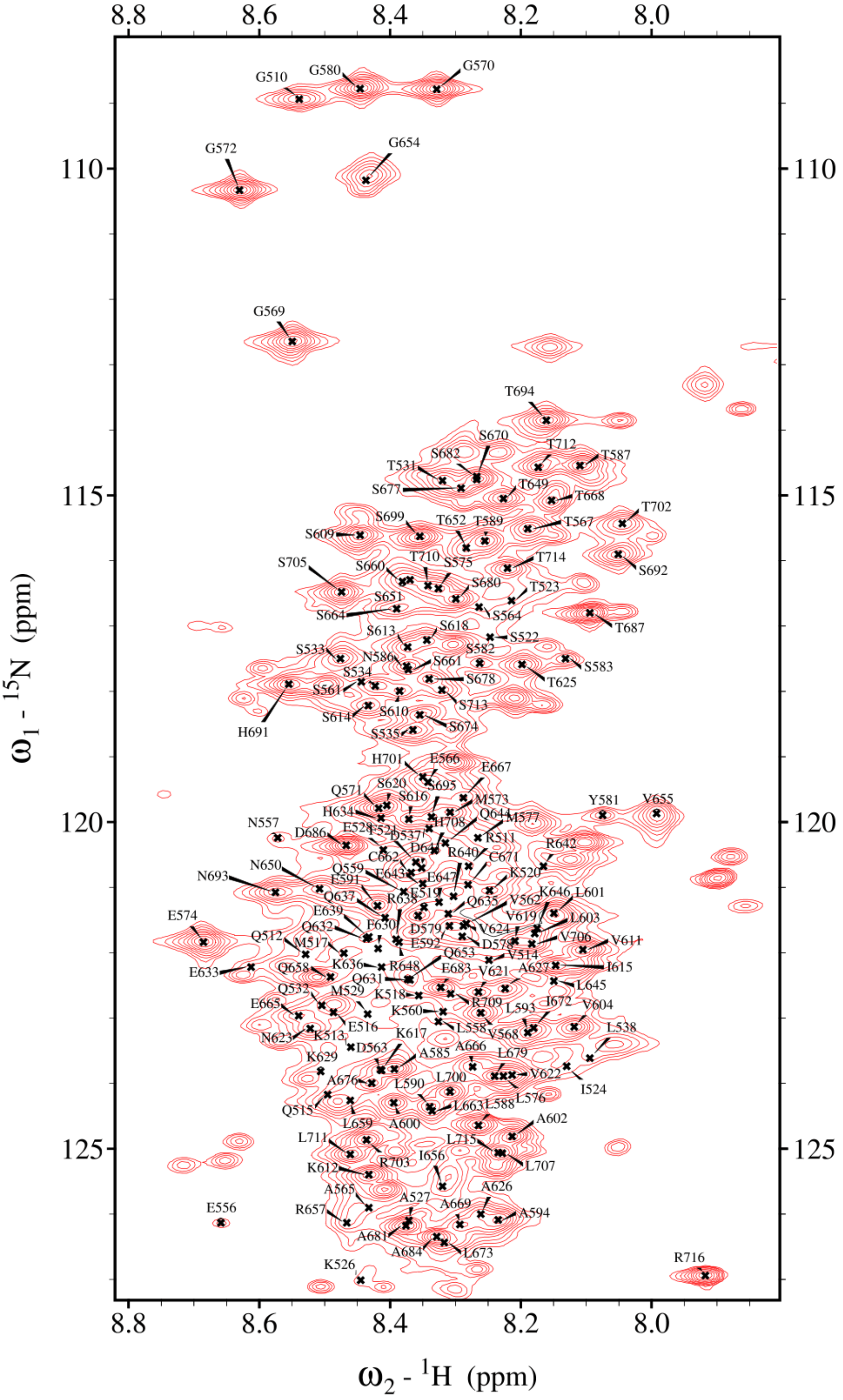

Figure S3. MetC assignment. *The 2D ^{15}N-HSQC spectrum of MetC presents features typical for IDPs with a very narrow chemical shift range (8-8.6 ppm in proton dimension). Most of observed signals strongly overlap. Single more dispersed signals (like 50GLU, 68GLU, 149VAL, 210ARG) may correspond to aa residues involved in the formation of local and transient motifs of the secondary structure (like MoREs). Assigned aa residues are indicated and signed with residue type and position in the protein sequence. The buffer used for MetC NMR analysis was 20 mM sodium phosphate pH 6.8, 50 mM NaCl, 1 mM TCEP, 0.5 mM EDTA.*

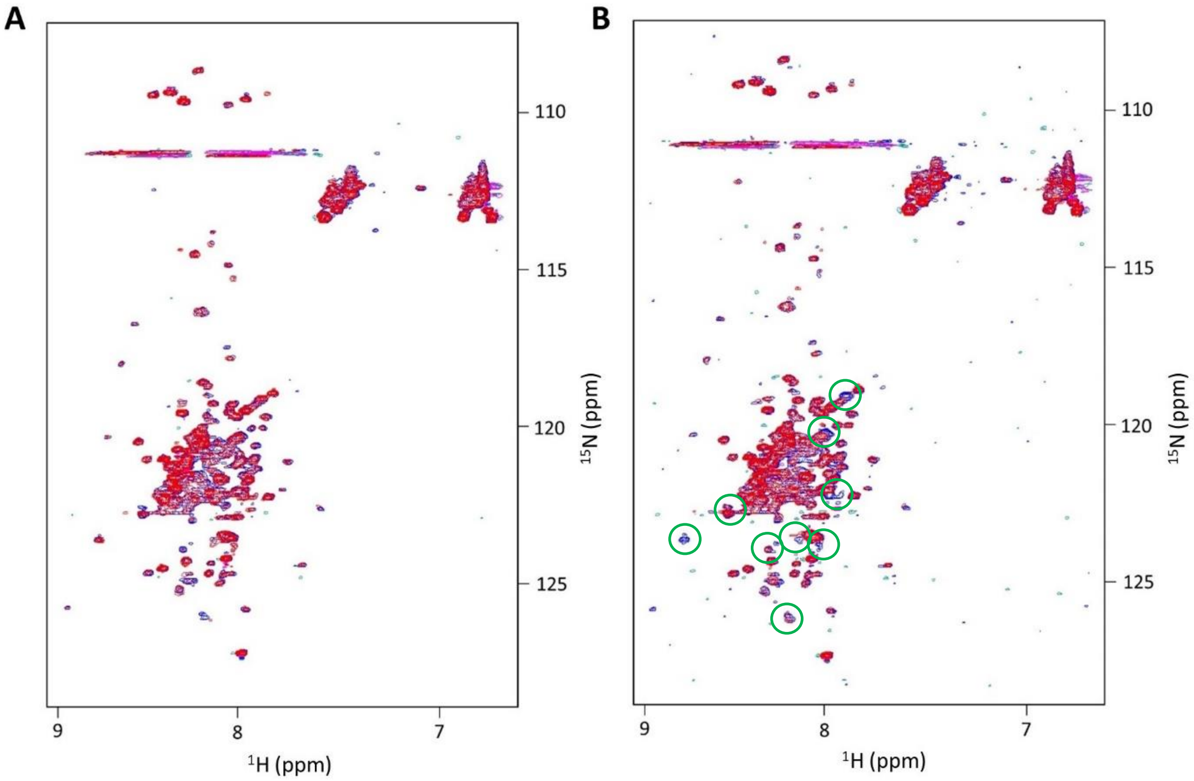

Figure S4. GceC chemical shift perturbation NMR spectrum. *A) GceC NMR reference spectrum (blue) and GceC spectrum obtained in the presence of equimolar concentration of Ftz-F1 LBD (red)* (Kolonko *et al.*, 2020). *Specific aa shifts are observed. B) P-GceC NMR reference spectrum (blue) and P-GceC spectrum obtained in the presence of equimolar concentration of 14-3-3 (red). Specific aa shifts are observed. The differences between two partners are indicated with green circles. The buffer used for GceC NMR analysis was PBS pH = 7.4, 2 mM DTT.*

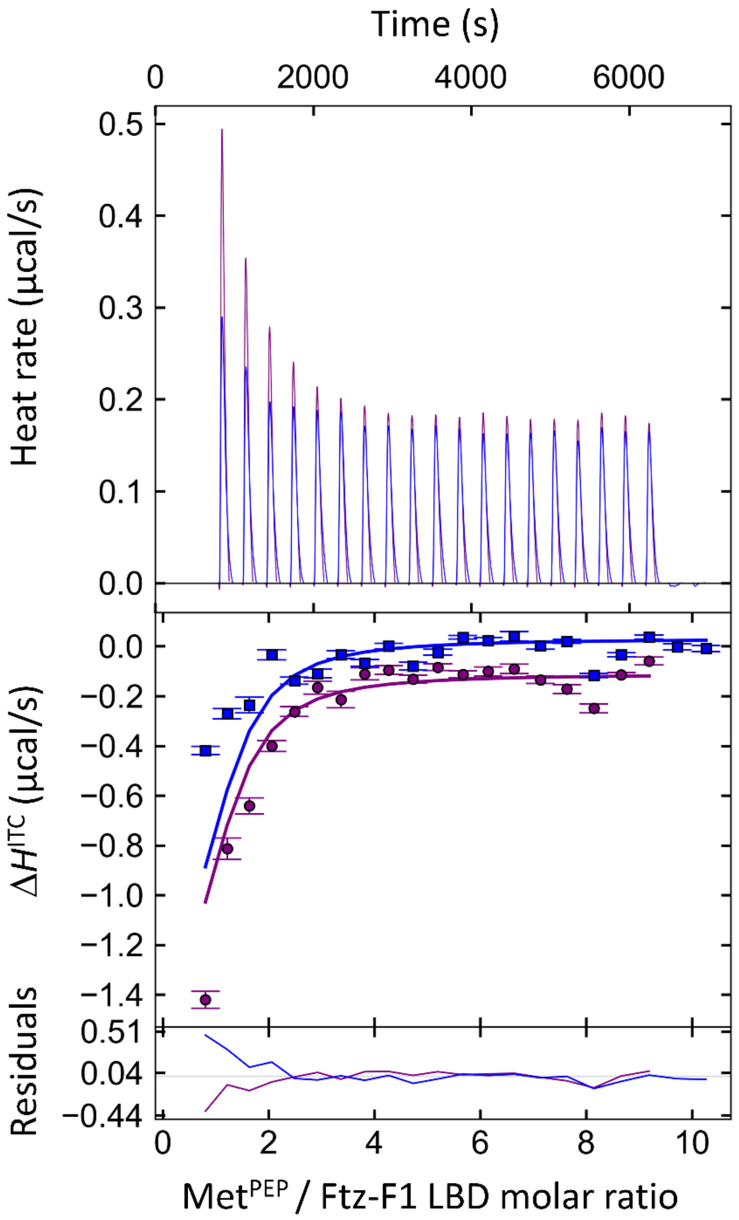

Figure S5. ITC profiles for Met^{PEP} binding to Ftz-F1 LBD. *The top panel shows the baseline-subtracted thermograms of two independent measurements (wine and blue lines). The bottom panel represents the binding isotherms together with error residuals. Data were fitted to independent model A + B ⇌ AB using global fitting mode. All measurements were obtained in 20 mM HEPES buffer, pH 6.8, 150 mM NaCl, and 1 mM TCEP.*

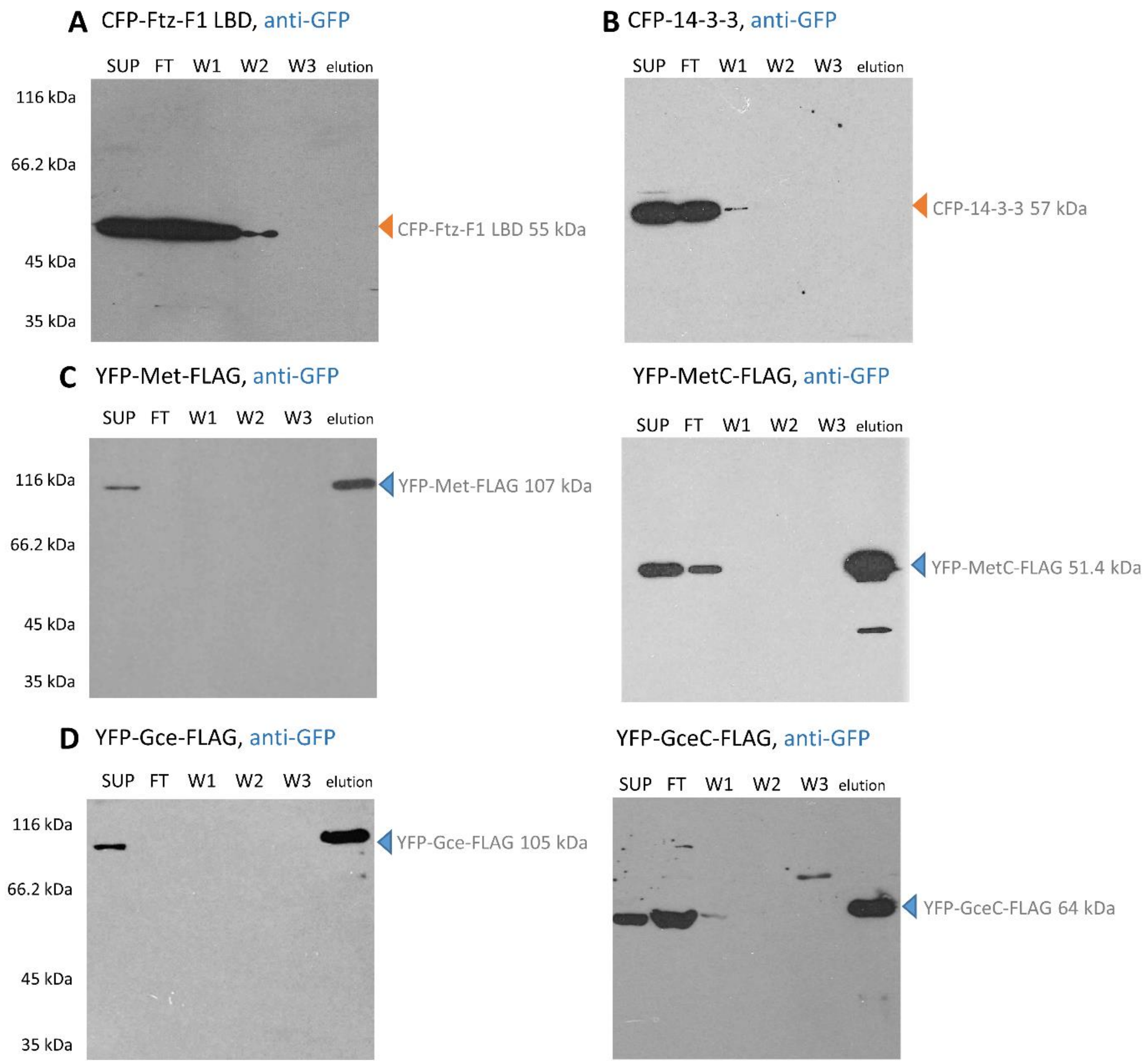

Figure S6. Pull-down experiment controls. *COS-7 cells were transfected with a vector encoding one protein (CFP-Ftz-F1 LBD, CFP-14-3-3, YFP-Met-FLAG, YFP-Gce-FLAG, YFP-MetC-FLAG, or YFP-GceC-FLAG) pulled down with ANTI-FLAG M2 Affinity gel. Finally, the samples were analysed for the presence of protein by Western blotting and detected by anti-GFP antibodies. A and B – negative controls, no bands are detected in the elution fraction. CFP-FTZ-F1 LBD and CFP-14-3-3 did not bind to the ANTI-FLAG M2 Affinity gel (marked with orange arrows). C and D – positive controls, a band corresponding to YFP-Met-FLAG, YFP-Gce-FLAG, YFP-MetC-FLAG, or YFP-GceC-FLAG it is present in the elution fraction for each protein (marked with blue arrows). Proteins tagged with FLAG have bound to the ANTI-FLAG M2 Affinity gel. SUP – supernatant; FT – proteins not bound to the resin; W1-3 – wash fraction; elution – elution fraction. Observed fusion proteins with MM are marked with arrows (blue for proteins with FLAG tag, binding to the ANTI-FLAG M2 Affinity gel and orange for untagged proteins, not binding to the ANTI-FLAG M2 Affinity gel). All experiments were repeated twice or more.*
